# Dialkylation of Indoles with Trichloroacetimidates to Access 3,3-Disubstituted Indolenines

**DOI:** 10.3390/molecules24224143

**Published:** 2019-11-15

**Authors:** Tamie Suzuki, Nilamber A. Mate, Arijit A. Adhikari, John D. Chisholm

**Affiliations:** Department of Chemistry, Syracuse University, 1-014 Center for Science and Technology, Syracuse, NY 13244-4100, USA; tsuzuki@southern.edu (T.S.); namate@syr.edu (N.A.M.); Arijit_Adhikari@hms.harvard.edu (A.A.A.)

**Keywords:** indole, dialkylation, indolenine, trichloroacetimidate, spiroindoline

## Abstract

2-Substituted indoles may be directly transformed to 3,3-dialkyl indolenines with trichloroacetimidate electrophiles and the Lewis acid TMSOTf. These reactions provide rapid access to complex indolenines which are present in a variety of complex natural products and medicinally relevant small molecule structures. This method provides an alternative to the use of transition metal catalysis. The indolenines are readily transformed into spiroindoline systems which are privileged scaffolds in medicinal chemistry.

## 1. Introduction

**Figure 1:**
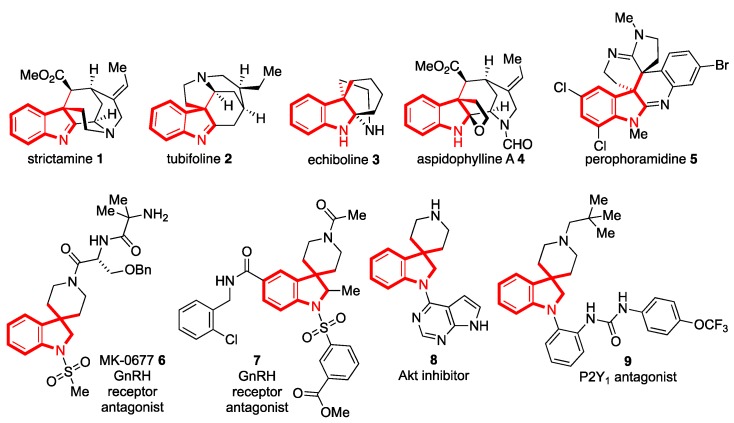
Natural products containing the 3,3-dialkyl indolenine motif and related spiroindoline structures.

3,3-Dialkyl indolenines are common substructures found in many complex alkaloids like strictamine **1** [1] and tubifoline **2** [2] (Figure 1). A number of other alkaloids appear to derive from the intramolecular addition of heteroatom nucleophiles to the indolenine. This includes complex alkaloids such as echiboline **3** [3], aspidophylline A **4** [4], and perophoramidine **5** [5] (Figure 1). 3,3-Dialkyl indolenines have also been utilized as platforms in medicinal chemistry studies [6,7], as a means to move towards more three-dimensional structures with a greater proportion of sp^3^ hybridized carbons, which is desirable in order to create molecules which interact with more complex pharmaceutical target receptors [8,9,10,11]. Structurally related spiropiperidine-indanes have also been referred to as “privileged scaffolds” [12,13,14] for the design of medicinally relevant small molecules, including the ghrelin receptor agonists MK-0677 **6** [15] and **7** [16], the Akt inhibitor **8** [17] and the P2Y_1_ antagonist **9** [18]. Besides their presence in natural products, similar indolines are also utilized as precursors to indolenine dyes [19], which have applications in biological imaging [20,21,22,23], sensors [24,25], and in solar cells [26,27].

Given the common nature of 3,3-dialkyl indolenines and related structures, researchers have been active in investigating efficient methods to access similar architectures [28,29,30,31]. These include intramolecular condensation of an aniline [32,33,34], the interrupted Fischer indole synthesis [35,36,37], and the addition of organometallic reagents to benzylic nitriles [38,39,40]. One popular method is the dearomatization of indoles [41,42,43,44] with an electrophilic alkylating agent. Many of these reactions are complicated by competing *N*-alkylation of the indole. In spite of this issue, a number of acid promoted [45,46,47], base promoted [48,49,50,51,52,53,54], and transition metal catalyzed [55,56,57,58,59,60,61,62,63,64] transformations have been described to access indolenines from 3-substituted indoles.

**Scheme 1:**
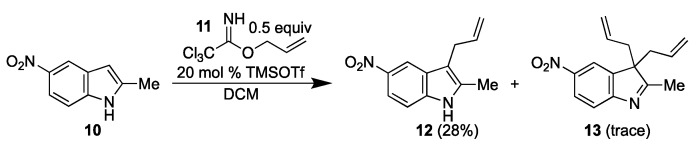
Detection of the dialkylation product **13** during alkylation of 5-nitro-2-methyl-indole **10.**

In a recent study on the alkylation of indoles utilizing trichloroacetimidate electrophiles [65], we observed a small amount of the dialkylated indolenine **13** as a side product from the TMSOTf catalyzed C3-alkylation of 2-methyl-5-nitroindole **10** with allyl imidate **11** in dichloromethane (DCM) (Scheme 1). While the formation of indolenines from 2,3-disubstituted indoles with imidates has been reported [66], the direct dialkylation of indoles could provide a rapid entry to 3,3-dialkyl indolenine intermediates from less substituted (and therefore less expensive) indole starting materials. This would provide an efficient alternative approach for the direct C3-dialkylation of indoles that does not rely on costly transition metal catalysts. The use of trichloroacetimidate electrophiles as the alkylating agent is attractive because they can be easily formed from readily available alcohols under mild conditions [67]. Intrigued by the potential of this dialkylation reaction, we began optimization studies to explore the scope of this Lewis acid promoted dearomatization reaction.

## 2. Results & Discussion

Our recent studies on promoter free substitution reactions with trichloroacetimidate electrophiles [68,69,70,71,72,73,74] led us to speculate that imidates may be reactive enough to participate in indole dialkylation without the need for a Lewis acid catalyst. Heating 2-methyl indole **14** and allyl trichloroacetimidate **11** in refluxing 1,2-dichloroethane (DCE) for 24 h showed no trace of alkylation product, however, so the use of TMSOTf as the Lewis acid was then investigated (Table 1). Previous investigations with indoles and trichloroacetimidates have demonstrated that TMSOTf is especially effective in these systems [65,66], and encouraging results were immediately obtained. Use of 20 mol% TMSOTf led to the formation of indolenine **15** with a 27% yield (Table 1, Entry 2). Increasing reaction time, temperature and using excess imidate were then evaluated, but these changes only led to modest increases in yield (Entries 3–5). Given that a more basic reaction media is being formed after the second alkylation (the imine on **15** is a functional base), it was considered that perhaps product inhibition was occurring, with the imine scavenging the Lewis acid and halting the reaction. An increase in the TMSOTf loading would therefore be necessary to obtain higher conversions. Increasing the amount of TMSOTf provided a 61% yield of **15** when a stoichiometric amount of the Lewis acid was employed (Entry 7). Further increasing the amount of TMSOTf did not significantly improve the yield, nor did heating the reaction. Little indole starting material **14** was isolated from the reaction, with the rest of the mass balance being a mixture of overalkylation products (alkylation can also occur at C5 and C7 of the indole ring).

The indole dialkylation was then evaluated with regard to the indole nucleophile. The addition of either electron donating or electron withdrawing groups to the 5-position of the indole was tolerated, with yields in the 40%–70% range being observed (Table 2). Interestingly, the 5-nitro-2-methyl indole **10** provided the diallylation product **13**, which is not accessible using palladium catalysis, as *N*-alkylation is favored when this indole is employed [6]. Changing the alkyl group at the 2-position of the indole was also explored. Use of indole (Entry 7) provided only a complex mixture of products, and this substrate was not pursued further. A more moderate yield was obtained with 2-phenylindole, likely due to steric effects from the larger group at the indole 2-position. Indole 2-carboxylic acid methyl ester **14h** was not reactive under these conditions, returning the starting indole and decomposed imidate from the reaction mixture. While many of these yields are moderate, it is important to realize that two reactions are actually occurring in sequence during the dialkylation, so the yield may perhaps be best thought of in terms of a sequence of two separate steps proceeding a ~75% yield where isolation and purification of the intermediate 3-alkylindole is avoided.

The efficacy of these conditions was then evaluated using a number of allylic and benzylic imidates (Table 3). More highly substituted allylic imidates gave lower yields, this may be due to the electrophile being more highly stabilized and therefore less reactive. Improved yields could be achieved by performing many of the reactions in refluxing DCE. Similar results were obtained with propargyl imidate **18**, which was less reactive (only providing trace product at room temperature) but would participate when the reaction was heated to reflux, albeit in a moderate yield. Benzylic trichloroacetimidates were also evaluated. The highly reactive 4-methoxybenzyl imidate **19** gave a complex mixture of products due to polyalkylation. Better results were obtained with the less reactive benzyl imidate **20**, which gave a 30% yield of the dialkylation product **15m** (38% when the reaction was performed under reflux). Benzylic imidates decorated with electron withdrawing groups (**21–23**) were also less reactive and provided only trace amounts of the dialkylation products at rt, with C3-monoalkylation being the major product [65]. Heating the reaction to reflux provided the desired dialkylation products in much improved overall yields, however.

**Scheme 2:**
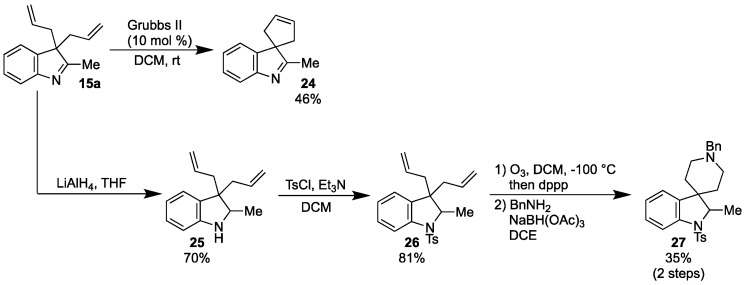
Elaboration of the 3,3-Diallyl indolenine **15a** to spiro-polycyclic systems.

With ready access to 3,3-diallyl indolenines via imidate alkylation, we turned our attention to the functionalization of these systems to three-dimensional scaffolds like those used in medicinal chemistry studies. Initially a spirocycle formation was explored utilizing the Grubbs metathesis catalyst. This led to the formation of spirocycle **24** (Scheme 2). The indolenine **15a** was also transformed into a spiropiperidine-indane that is similar to that found in the ghrelin receptor agonists MK-0677 **6** and **7**. This involved initial reduction of the indolenine **15a** to the indoline **25** with lithium aluminum hydride. The sulfonamide **26** was then formed with TsCl and triethylamine. Oxidative cleavage of the alkenes to the corresponding aldehyde was executed via ozonolysis. Purification of this dialdehyde proved difficult when triphenylphosphine was used to reduce the ozonide, but the use of 1,3-bis(diphenylphosphino)propane (dppp) as the reductant made the purification easier as the bisphosphine oxide was more polar and easier to separate from the product. The dialdehyde proved to be unstable and readily self-condensed, so it was immediately subjected to a reductive amination with benzylamine and NaBH(OAc)_3_, which provided the desired spiropiperidine-indane **27** with a 35% yield over two steps.

**Figure 2:**
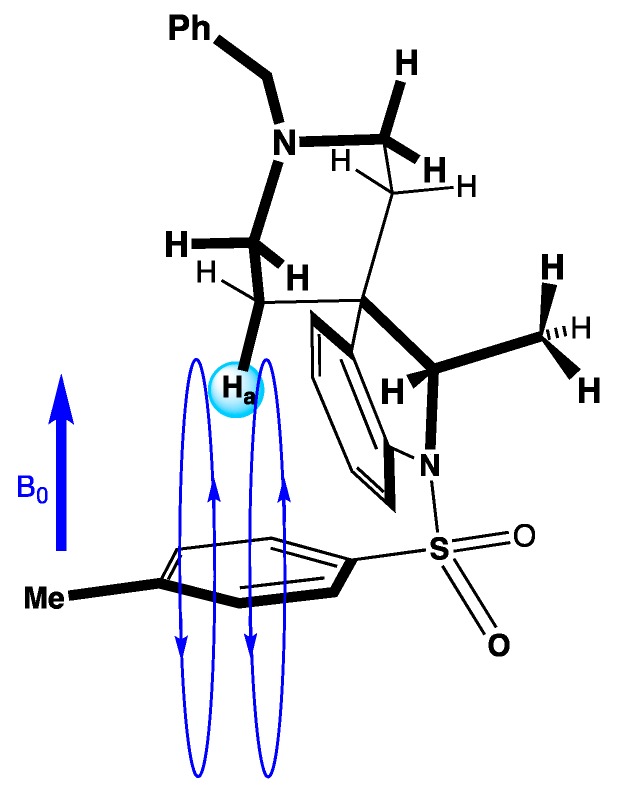
Upfield Shift of Ha in the ^1^H NMR of Spiropiperidine **27** due to Diamagnetic Anisotropy from the Sulfonamide -System.

Oddly, the spiropiperidine **27** showed a multiplet in the ^1^H NMR at 0.28 ppm that integrated for a single hydrogen resonance. A proton with this chemical shift was not congruent with the proposed structure, so some additional studies were performed. A COSY experiment verified that the upfield proton was part of the piperidine ring. Some molecular modeling studies indicated that this unusual chemical shift is likely to be attributed to diamagnetic anisotropy from the aromatic ring of the toluenesulfonamide, which prefers to reside on the opposite face of the pyrroline ring as the methyl group due to steric effects. This holds the -system of the sulfonamide in a position to shield one of the protons on the piperidine ring (H_a_, Figure 2). The molecular modeling predicts that in the lowest energy conformation H_a_ is only ~2.8 Å from the center of the aromatic ring. This upfield chemical shift is consistent with literature reports of similar spiropiperidine-indanes [75]. In further support of this rationale, in structures where the C2 position of the pyrroline is unsubstituted [76], or there is no aromatic sulfonamide [77], no similar upfield shifts are observed in the ^1^H NMR.

## 3. Materials and Methods

### 3.1. General Experimental Information

All anhydrous reactions were run under a positive pressure of argon. Dichloromethane (DCM) was dried by passage through an alumina column. 1,2-Dichloroethane (DCE) was freshly distilled from calcium hydride before use. Silica gel column chromatography was performed using 60 Å silica gel (230−400 mesh). Melting points are uncorrected. The indoles used in this study were purchased from commercial sources.

### 3.2. Preparation of Trichloroacetimidates

Allyl-2,2,2-trichloroacetimidate **11** [78], 1-(1-imino-2,2,2-trichloroethoxy)-3-phenyl-2(E)-propene **17** [79], propargyl-2,2,2-trichloroacetimidate **18** [70], (4-methoxyphenyl)methyl-2,2,2-trichloroacetimidate **19** [69], benzyl-2,2,2-trichloroacetimidate **20** [78], (4-chloro)methyl-2,2,2-trichloroacetimidate **21** [80], (4-trifluoromethyl)methyl-2,2,2-trichloroacetimidate **22** [66], and (4-nitrophenyl)methyl-2,2,2-trichloroacetimidate **23** [81] were synthesized as previously reported.

2-Methyl-2-propenyl trichloroacetimidate (**16**). A flame dried flask was charged with 2-methyl-2-propen-1-ol (7.0 mmol, 0.589 mL) and placed under argon. Dry DCM (35 mL) was then added, and the flask was cooled to 0 °C. 1,8-Diazabicyclo [5.4.0]undec-7-ene (0.7 mmol, 0.108 mL) was added to the solution, followed by trichloroacetonitrile (8.4 mmol, 0.843 mL). After ~22 h the reaction mixture was concentrated and the residue purified by silica gel column chromatography (10% EA/3% Et_3_N/87% hexanes). Clear oil (1.502 g, 99%). TLC Rf = 0.42 (60% DCM/40% hexanes); IR (thin film) 3365, 3072, 2975, 2904, 1637, 1607, 1482, 912 cm^−1^; ^1^H NMR (400 MHz, CDCl_3_) δ 8.31 (bs, 1H), 5.11 (s, 1H), 4.99 (s, 1H), 4.71 (s, 2H), 1.83 (s, 3H); ^13^C NMR (100 MHz, CDCl_3_) δ 162.6, 139.4, 113.3, 91.5, 72.3, 19.4.; HRMS (ESI+) calcd for C_6_H_8_Cl_3_NONa^+^ [M + Na]^+^: 237.9563. Found: 237.9564.

### 3.3. Synthesis of 3,3′-Disubstituted Indolenines

General procedure for C3-dialkylation of indoles. In a flame dried flask, the imidate (2.5 equiv) was dissolved in anhydrous DCE (0.3 M) followed by the addition of the indole (1.0 equiv). To this solution freshly distilled TMSOTf (1.0 equiv) was added and the resulting mixture was stirred at room temp. or heated to reflux for 3 h. After cooling to room temperature, the reaction mixture was quenched with 10 mL 1 M NaOH. The organic layer was separated and the aqueous layer was extracted with DCM (3 × 5 mL). The combined organic layers were dried over sodium sulfate, filtered and concentrated. The residue was purified by silica gel chromatography using the listed solvent system.

3,3-Diallyl-2-methyl-3H-indole (**15a**). Synthesized by the general procedure from 2-methylindole **14a** and imidate **11** [78], purified using silica gel chromatography (3% EA/97% DCM). This compound has been previously reported [58]. Orange oil (0.14 g, 59%); TLC R_f_ = 0.35 (5% EA/95% DCM); ^1^H NMR (300 MHz, CDCl_3_) δ 7.52 (d, *J* = 7.6 Hz, 1H), 7.34–7.28 (m, 2H), 7.22–1.17 (m, 1H), 5.18–5.05 (m, 2H), 4.95 (d, *J* = 17.0 Hz, 2H), 4.85 (d, *J* = 10.9 Hz, 2H), 2.69 (dd, *J* = 13.9, 6.1 Hz, 2H), 2.45 (dd, *J* = 13.9, 7.7 Hz, 2H), 2.25 (s, 3H); ^13^C NMR (100 MHz, CDCl_3_) δ 185.2, 154.8, 141.2, 132.1, 127.9, 125.0, 122.2, 119.8, 118.1, 61.8, 40.3, 16.5.

3,3-Diallyl-5-methoxy-2-methyl-3H-indole (**15b**). Synthesized by general procedure from 5-methoxy-2-methyl-1*H*-indole **14b** and imidate **11** [78], purified using silica gel chromatography (5% EA/95% DCM). This compound has been previously reported [6]. Brown solid (0.18 g, 61%); mp = 45–46 °C; TLC Rf = 0.33 (10% EA/90% DCM); IR (ATR) 3077, 3000, 1640, 1591, 1576, 908 cm^−1^; ^1^H NMR (300 MHz, CDCl_3_) δ 7.44 (d, *J* = 9.0 Hz, 1H), 6.85–6.83 (m, 2H), 5.20–5.06 (m, 2H), 4.96 (d, *J* = 16.2 Hz, 2H), 4.87 (d, *J* = 9.8 Hz, 2H), 3.83 (s, 3H), 2.66 (dd, *J* = 13.8, 5.8 Hz, 2H), 2.45 (dd, *J* = 13.9, 7.7 Hz, 2H), 2.25 (s, 3H); ^13^C NMR (100 MHz, CDCl_3_) δ 182.9, 157.8, 142.9, 132.1, 119.9, 118.1, 117.5, 112.2, 109.2, 61.9, 55.7, 40.4, 16.4.

3,3-Diallyl-2,5-dimethyl-3H-indole (**15c**). Synthesized by general procedure from 2,5-dimethyl-1*H*-indole **14c** and imidate **11** [78], purified using silica gel chromatography (20% EA/80% hexanes). Brown solid (0.13 g, 41%); mp = 38–40 °C; TLC Rf = 0.38 (40% EA/60% hexanes); IR (ATR) 3081, 3002, 1638, 1574, 820 cm^−1^; ^1^H NMR (400 MHz, CDCl_3_) δ 7.38 (d, *J* = 7.8 Hz, 1H), 7.11 (d, *J* = 7.8 Hz, 1H), 7.07 (s, 1H), 5.16–5.06 (m, 2H), 4.95 (d, *J* = 17.0 Hz, 2H), 4.85 (d, *J* = 9.9 Hz, 2H), 2.66 (dd, *J* = 13.9, 6.0 Hz, 2H), 2.43 (dd, *J* = 13.9, 7.8 Hz, 2H), 2.39 (s, 3H), 2.22 (s, 3H); ^13^C NMR (100 MHz, CDCl_3_) δ 184.0, 152.7, 141.3, 134.7, 132.3, 128.5, 122.9, 119.3, 118.0, 61.5, 40.4, 21.5, 16.5; HRMS (ESI+) m/z calcd for C_16_H_19_NNa^+^ [M + Na]^+^: 248.1409, found: 248.1409.

3,3-Diallyl-5-chloro-2-methyl-3H-indole (**15d**). Synthesized by general procedure from 5-chloro-2-methyl-1*H*-indole **14d** and imidate **11** [78], purified using silica gel chromatography (10% EA/90% hexanes). Yellow oil (0.10 g, 45%); TLC Rf = 0.42 (30% EA/70% hexanes); IR (ATR) 3076, 1728, 1577, 1451, 920, 825 cm^−1^; ^1^H NMR (400 MHz, CDCl_3_) δ 7.44 (d, *J* = 8.4 Hz, 1H), 7.31–7.26 (m, 2H), 5.18–5.08 (m, 2H), 4.99 (d, *J* = 16.8 Hz, 2H), 4.91 (d, *J* = 10.4 Hz, 2H), 2.68 (dd, *J* = 13.6, 6.0 Hz, 2H), 2.47 (dd, *J* = 14.0, 7.6 Hz, 2H), 2.26 (s, 3H); ^13^C NMR (100 MHz, CDCl_3_) δ 185.7, 153.4, 143.1, 131.5, 130.9, 128.1, 122.7, 120.6, 118.6, 62.3, 40.1, 16.5; HRMS (ESI+) m/z calcd for C_15_H_16_ClNNa^+^ [M + Na]^+^: 268.0863, found: 268.0864.

3,3-Diallyl-5-fluoro-2-methyl-3H-indole (**15e**). Synthesized by general procedure from 5-chloro-2-methyl-1H-indole **14e** and imidate **11** [78], purified using silica gel chromatography (10% EA/90% hexanes). Purple oil (0.16 g, 68%); TLC Rf = 0.37 (30% EA/70% hexanes); IR (ATR) 3077, 1727, 1581, 1462, 918, 821 cm^−1^; ^1^H NMR (400 MHz, CDCl_3_) δ 7.41 (dd, *J* = 8.2, 4.7 Hz, 1H), 7.00–6.94 (m, 2H), 5.15–5.04 (m, 2H), 4.94 (d, *J* = 16.8 Hz, 2H), 4.85 (d, *J* = 10.0 Hz, 2H), 2.63 (dd, *J* = 13.9, 6.3 Hz, 2H), 2.43 (dd, *J* = 13.6, 7.7 Hz, 2H), 2.21 (s, 3H); ^13^C NMR (100 MHz, CDCl_3_) δ 184.8 (d, *J* = 3.5 Hz), 161.0 (d, *J* = 242.1 Hz), 150.8 (d, *J* = 1.8 Hz), 143.2 (d, *J* = 8.5 Hz), 131.6, 120.3 (d, *J* = 8.8 Hz), 118.5, 114.6 (d, *J* = 23.4 Hz), 109.9 (d, *J* = 23.3 Hz), 62.3 (d, *J* = 2.0 Hz), 40.2, 16.4; HRMS (ESI+) m/z calcd for C_15_H_16_FNNa^+^ [M + Na]^+^: 252.1159, found: 252.1158.

3,3-Diallyl-5-nitro-2-methyl-3H-indole (**13**). Synthesized by the general procedure from 2-methyl-5-nitro-1*H*-indole **10** and imidate **11** [78], purified using silica gel chromatography (5% EA/95% DCM). Brown oil (0.21 g, 71%); TLC Rf = 0.47 (10% EA/90% DCM); IR (ATR) 3007, 1703, 1571, 1518, 1338 cm^−1^; ^1^H NMR (300 MHz, CDCl_3_) δ 8.28 (dd, *J* = 8.5, 2.3 Hz, 1H), 8.16 (d, *J* = 2.0 Hz, 1H), 7.62 (d, *J* = 8.5 Hz, 1H), 5.17–5.04 (m, 2H), 5.03–4.88 (m, 4H), 2.77 (dd, *J* = 14.0, 6.1 Hz, 2H), 2.53 (dd, *J* = 14.0, 7.1 Hz, 2H), 2.35 (s, 3H); ^13^C NMR (100 MHz, CDCl_3_) δ 191.6, 159.6, 145.6, 142.4, 130.7, 124.9, 119.9, 119.4, 117.9, 62.9, 40.0, 17.0; HRMS (ESI+) m/z calcd for C_15_H_16_N_2_O_2_Na^+^ [M + Na]^+^: 279.1104, found: 279.1103.

3,3-Diallyl-2-phenyl-3H-indole (**15g**). Synthesized by general procedure from the known indole **14g** and imidate **11** [78], purified using silica gel chromatography (5% EA/95% hexanes). This compound has been previously reported [6]. Yellow oil (0.09 g, 34%); TLC Rf = 0.52 (5% EA/95% DCM); ^1^H NMR (300 MHz, CDCl_3_) δ 8.14–8.10 (m, 2H), 7.67 (d, *J* = 7.5 Hz, 1H), 7.49–7.47 (m, 3H), 7.40–7.26 (m, 3H), 5.18–5.05 (m, 2H), 4.79–4.71 (m, 4H), 2.90 (d, *J* = 6.9 Hz, 4H); ^13^C NMR (100 MHz, CDCl_3_) δ 180.3, 154.4, 142.9, 133.9, 131.8, 130.6, 128.6, 128.1, 128.0, 125.7, 121.7, 120.7, 118.3, 62.4, 41.8.

2-Methyl-3,3-bis(2-methyl-2-propenyl)-3H-indole (**15i**). Synthesized by general procedure from 2-methylindole **14a** and imidate **16**, purified using silica gel chromatography (10% EA/90% hexanes). Yellow oil (0.13 g, 46%); TLC Rf = 0.52 (5% EA/95% DCM); IR (ATR) 3074, 2967, 2918, 1642, 1575,1447, 765 cm^−1^; ^1^H NMR (400 MHz, CDCl_3_) δ 7.42 (d, *J* = 8.0 Hz, 1H), 7.25–7.21 (m, 2H), 7.12–7.09 (m, 1H), 4.48–4.47 (m, 2H), 4.40 (s, 2H), 2.63 (d, *J* = 13.6 Hz, 2H), 2.53 (d, *J* = 14.0 Hz, 2H), 2.26 (s, 3H), 1.06 (s, 6H); ^13^C NMR (100 MHz, CDCl_3_) δ 185.8, 155.2, 141.7, 140.9, 127.8, 124.6, 122.9, 120.0, 114.2, 62.3, 45.5, 23.4, 17.2; HRMS (ESI+) m/z calcd for C_17_H_21_NNa^+^ [M + Na]^+^: 262.1566, found: 262.1566.

3,3-Bis[(E)-3-phenyl-2-propenyl]-2-methyl-3H-indole (**15j**). Synthesized by the general procedure from 2-methylindole **14a** and imidate **17** [79], purified using silica gel chromatography (100% DCM). Yellow oil (0.055 g, 20%); TLC Rf = 0.26 (100% DCM); IR (ATR) 3024, 2919, 1576, 1447, 906, 730 cm^−1^; ^1^H NMR (400 MHz, CDCl_3_) δ 7.53 (d, *J* = 7.6 Hz, 1H), 7.35–7.31 (m, 3H), 7.26–7.12 (m, 10H), 6.35 (d, *J* = 15.7 Hz, 2H), 5.60–5.52 (m, 2H), 2.89 (dd, *J* = 14.0, 6.7 Hz, 2H), 2.63 (dd, *J* = 13.9, 8.0 Hz, 2H), 2.33 (s, 3H); ^13^C NMR (100 MHz, CDCl_3_) δ 185.0, 154.8, 141.2, 136.9, 133.3, 128.5, 128.4, 128.1, 127.3, 126.1, 125.1, 123.7, 122.3, 120, 62.1, 39.4, 16.7; HRMS (ESI+) m/z calcd for C_27_H_25_NNa^+^ [M + Na]^+^: 386.1879, found: 386.1878.

2-Methyl-3,3-di(prop-2-yn-1-yl)-3H-indole (**15k**). Synthesized by the general procedure from 2-methylindole **14a** and imidate **18** [70], purified using silica gel chromatography (3% EA/97% DCM). Yellow oil (0.06 g, 24%); TLC Rf = 0.55 (10% EA/90% DCM); IR (ATR) 3285, 2924, 2119, 1579, 1468, 770, 624 cm^−1^; ^1^H NMR (400 MHz, CDCl_3_) δ 7.53 (t, *J* = 7.7 Hz, 2H), 7.36 (dt, *J* = 7.6, 1.2 Hz, 1H), 7.22 (t, *J* = 7.4 Hz, 1H), 2.76 (dd, *J* = 16.7, 2.6 Hz, 2H), 2.60 (dd, *J* = 16.8, 2.6 Hz, 2H), 2.37 (s, 3H), 1.98 (t, *J* = 2.6 Hz, 2H); ^13^C NMR (100 MHz, CDCl_3_) δ 183.1, 154.4, 140.0, 128.7, 125.4, 122.4, 120.0, 78.6, 71.5, 57.6, 24.4, 16.7; HRMS (ESI+) m/z calcd for C_15_H_13_NNa^+^ [M + Na]^+^: 230.0940, found: 230.0939.

3,3-Dibenzyl-2-methyl-3H-indole (**15m**). Synthesized by the general procedure from 2-methylindole **14a** the imidate **20** [78], purified using silica gel chromatography (1% EA/99% DCM). This compound has been previously reported [58]. Brown oil (0.10 g, 30%); TLC R_f_ = 0.49 (5% EA/95% DCM); ^1^H NMR (400 MHz, CDCl_3_) δ 7.18–7.14 (m, 2H), 7.06 (td, *J* = 7.2, 1.6 Hz, 1H), 7.02–6.95 (m, 7H), 6.68 (dd, *J* = 7.2, 1.2 Hz, 4H), 3.28 (d, *J* = 13.6 Hz, 2H), 2.99 (d, *J* = 13.6 Hz, 2H), 2.32 (s, 3H); ^13^C NMR (100 MHz, CDCl_3_) δ 184.1, 155.1, 140.5, 135.7, 129.4, 127.9, 127.8, 126.7, 124.4, 23.7, 119.8, 64.0, 42.3, 17.3.

3,3-Bis[(p-chlorophenyl)methyl]-2-methyl-3H-indole (**15n**). Synthesized by the general procedure from 2-methylindole **14a** and imidate **21** [80], purified using silica gel chromatography (3% EA/97% DCM). Yellow oil (0.08 g, 18%); TLC Rf = 0.24 (30% EA/70% hexanes); IR (ATR) 3046, 2918, 2848, 1595, 1491, 1013, 837 cm^−1^; ^1^H NMR (300 MHz, CDCl_3_) δ 7.32–7.17 (m, 3H), 7.11 (d, *J* = 6.9 Hz, 1H), 7.02 (d, *J* = 8.4 Hz, 4H), 6.66 (d, *J* = 8.4 Hz, 4H), 3.32 (d, *J* = 13.7 Hz, 2H), 3.04 (d, *J* = 13.6 Hz, 2H), 2.42 (s, 3H); ^13^C NMR (100 MHz, CDCl_3_) δ 183.3, 155.1, 139.8, 133.9, 132.7, 130.5, 128.3, 128.0, 124.6, 123.3, 120.2, 63.8, 41.5, 17.3; HRMS (ESI+) m/z calcd for C_23_H_19_Cl_2_NNa^+^ [M + Na]^+^: 402.0787, found: 402.0785.

2-Methyl-3,3-bis{[p-(trifluoromethyl)phenyl]methyl}-3H-indole (**15o**). Synthesized by the general procedure from the known indole **14a** and imidate **22** [66], purified using silica gel chromatography (2% EA/98% DCM). Yellow oil (0.23 g, 45%); TLC Rf = 0.51 (5% EA/95% DCM); IR (ATR) 3049, 2924, 1919, 1726, 1616, 1319, 1100 cm^−1^; ^1^H NMR (400 MHz, CDCl_3_) δ 7.30 (d, *J* = 8.4 Hz, 4H), 7.26–7.19 (m, 3H), 7.15 (d, *J* = 7.2 Hz, 1H), 6.84 (d, *J* = 8.0 Hz, 4H), 3.42 (d, *J* = 13.6 Hz, 2H), 3.15 (d, *J* = 13.6 Hz, 2H), 2.40 (s, 3H); ^13^C NMR (100 MHz, CDCl_3_) δ 182.8, 154.9, 139.4, 129.5, 129.4 (q, *J* = 32.3 Hz), 128.6, 124.8 (q, *J* = 3.7 Hz), 124.0 (q, *J* = 270.3 Hz), 123.2, 120.3, 63.6, 42.0, 17.2; HRMS (ESI+) m/z calcd for C_25_H_19_F_6_NNa^+^ [M + Na]^+^: 470.1314, found: 470.1311.

2-Methyl-3,3-bis[(p-nitrophenyl)methyl]-3H-indole (**15p**). Synthesized by general procedure from 2-methylindole **14a** and imidate **23** [81], purified using silica gel chromatography (50% EA/50% hexanes). Yellow oil (0.29 g, 63%); TLC Rf = 0.50 (10% EA/90% DCM); IR (ATR) 3076, 1728, 1577, 1451, 920, 825 cm^−1^; ^1^H NMR (400 MHz, CDCl_3_) δ 7.90 (d, *J* = 8.8 Hz, 4H), 7.31–7.20 (m, 4H), 6.88 (d, *J* = 8.4 Hz, 4H), 3.48 (d, *J* = 13.6 Hz, 2H), 3.26 (d, *J* = 13.6 Hz, 2H), 2.44 (s, 3H); ^13^C NMR (100 MHz, CDCl_3_) δ 182.0, 154.9, 147.0, 142.6, 138.7, 129.9, 129.0, 125.3, 123.1, 123.0, 120.6, 63.6, 41.9, 17.2; HRMS (ESI+) m/z calcd for C_23_H_19_N_3_O_4_ [M + H]^+^: 402.1448, found: 402.1451.

### 3.4. Elaboration of the 3,3′-Disubstituted Indolenines

2’-Methylspiro[3-cyclopentene-1,3’-indole] (**24**). The diallyl indoline **15a** (0.236 mmol, 50 mg) was dissolved in 2 mL of DCM. In a round bottom flask, Grubbs II catalyst (0.024 mmol, 21 mg) was taken in DCM (4 mL) and flushed with argon. The indoline in DCM was then added dropwise to the flask and stirred for 6 h at rt. Evaporated the solvent, purified using silica gel chromatography (5% EA/95% DCM). This compound has been previously prepared [7]. Yellow oil (21 mg, 46%); TLC Rf = 0.43 (10% EA/90% DCM); ^1^H NMR (400 MHz, CDCl_3_) δ 7.52 (d, *J* = 7.6 Hz, 1H), 7.34 (d, *J* = 7.6 Hz, 1H), 7.29 (dt, *J* = 7.6, 0.8 Hz, 1H), 7.18 (t, *J* = 7.6 Hz, 1H), 5.92 (s, 2H), 2.68 (s, 4H), 2.27 (s, 3H); ^13^C NMR (100 MHz, CDCl_3_) δ 186.9, 153.7, 146.4, 129.8, 127.6, 125.5. 121.0, 119.5, 61.4, 41.4, 15.6.

3,3-Diallyl-2-methylindoline (**25**). The diallyl indoline **15a** (2.37 mmol, 0.50 g) was dissolved in 10 mL THF and cooled to 0 °C using an ice bath. LiAlH_4_ solution (1 M in THF, 8.3 mmol, 8.3 mL) was then slowly added. After 5 min the reaction mixture was allowed to warm to room temperature. After 30 min the reaction mixture was recooled to 0 °C and quenched by dropwise addition of 15 mL solution of saturated aqueous Rochelle’s salt (potassium sodium tartrate). The reaction was poured into another 15 mL solution of saturated aqueous Rochelle’s salt and extracted to with EA (3 × 20 mL). The organic layers were combined and washed with saturated aqueous sodium chloride (50 mL), dried over sodium sulfate, filtered and concentrated. Purification using silica gel chromatography (60% DCM/40% hexanes) provided indoline **25**. Colorless oil (0.35 g, 70%); TLC Rf = 0.42 (60% DCM/40% hexanes); IR (ATR) 3365, 3072, 2975, 2904, 1637, 1607, 1482, 912 cm^−1^; ^1^H NMR (400 MHz, CDCl_3_) δ 7.04 (dt, *J* = 7.6, 1.2 Hz, 1H), 6.99 (d, *J* = 7.4 Hz, 1H), 6.74 (dt, *J* = 7.4, 0.8 Hz, 1H), 6.65 (d, *J* = 7.8 Hz, 1H), 5.80–5.68 (m, 2H), 5.06–4.97 (m, 4H), 3.78 (q, *J* = 6.6 Hz, 1H), 2.53 (dd, *J* = 14.2, 6.6 Hz, 1H), 2.39 (dd, *J* = 14.0, 7.9 Hz, 2H), 2.16 (dd, *J* = 13.8, 7.8 Hz, 1H), 1.24 (d, *J* = 6.6 Hz, 3H); ^13^C NMR (100 MHz, CDCl_3_) δ 149.5, 134.92, 134.91, 134.6, 127.5, 124.4, 118.6, 117.7, 117.5, 109.7, 62.5, 49.3, 40.5, 37.5, 15.3. HRMS (ESI+) calcd for C_15_H_19_NH^+^ [M + H]^+^: 214.1590, found: 214.1594.

3,3-Diallyl-2-methyl-1-(p-tolylsulfonyl)indoline (**26**). The diallyl indole **25** (5.02 mmol, 1.07 g) was dissolved in 20 mL of DCM and *p*-toluene sulfonyl chloride (8.78 mmol, 1.67 g) was added followed by triethylamine (10.97 mmol, 1.52 mL). After 16 h the reaction was quenched with 1M aq. HCl (50 mL) and extracted with DCM (3 × 50 mL). The combined organic extracts were washed with sat. aq. NaCl (50 mL), dried over Na_2_SO_4_, filtered and concentrated. Purification using silica gel chromatography (70% DCM/30% hexanes) provided sulfonamide **26**. Colorless oil (1.49 g, 81%); TLC Rf = 0.48 (80% DCM/20% hexanes); IR (ATR) 3073, 2979, 1637, 1457,1351, 1163, 915 cm^−1^; ^1^H NMR (300 MHz, CDCl_3_) δ 7.70–7.67 (m, 3H), 7.25–7.19 (m, 3H), 6.98–6.96 (m, 2H), 5.80–5.66 (m, 1H), 5.41–5.27 (m, 1H), 5.11 (s, 1H), 5.07 (d, *J* = 7.6 Hz, 1H), 4.83 (d, *J* = 13.6 Hz, 1H), 4.59 (d, *J* = 16.8 Hz, 1H), 3.99 (q, *J* = 6.6 Hz, 1H), 2.49 (dd, *J* = 14.9, 7.7 Hz, 1H), 2.35 (s, 3H), 2.30 (dd, *J* = 14.8, 6.3 Hz, 1H), 1.92 (dd, *J* = 13.9, 7.2 Hz, 1H) 1.46–1.39 (m, 4H); ^13^C NMR (100 MHz, CDCl_3_) δ 143.9, 140.2, 136.5, 135.6, 133.9, 132.7, 129.5, 128.2, 127.0, 124.6, 123.2, 118.7, 118.6, 115.5, 67.4, 49.6, 42.8, 36.3, 21.5, 17.5. HRMS (ESI+) calcd for C_22_H_25_NO_2_SNa^+^ [M + Na]^+^: 390.1498, found: 390.1495.

1’-Benzyl-2-methyl-1-(p-tolylsulfonyl)spiro[indoline-3,4’-piperidine] (**27**). The diallyl indole **26** (0.517 mmol, 0.190 g) was dissolved in 5 mL DCM and cooled to −100 °C (dry ice/ethyl ether bath). Ozone was then bubbled through the solution for about 2 min until the color changed to blue. The reaction mixture was then purged with argon until the blur color dissipated. 1,3-Bis(diphenylphosphino)propane (0.517 mmol, 0.213 g) was then added and reaction mixture was allowed to warm to room temp. The reaction mixture was then stirred for 1.5 h and then the solvent was evaporated. The resulting residue was purified by silica gel chromatography (75%EA/25%DCM) to provide the corresponding crude dialdehyde. The dialdehyde was dissolved in DCE (5.3 mL) and benzylamine (0.269 mmol, 0.029 mL) was added. After 5 min, sodium triacetoxyborohydride (1.07 mmol, 0.227 g) was added. After 16 h, the reaction was quenched with addition of water (5 mL) and extracted with DCM (3 × 10 mL). The combined organic extracts were washed with sat. aq. NaCl (20 mL), dried over sodium sulfate and concentrated. Purification of the residue using silica gel chromatography (20% EA/80% DCM) provided piperidine **27**. Off-white powder (0.08 g, 35% over 2 steps); TLC Rf = 0.47 (80% DCM/20%EA); IR (ATR) 2927, 2802, 2757, 2359, 2341, 1598, 1493, 1348, 1132 cm^−1^; ^1^H NMR (400 MHz, CDCl_3_) δ 7.67 (d, *J* = 8.2 Hz, 2H), 7.64 (d, *J* = 8.0 Hz, 1H), 7.33–7.28 (m, 5H), 7.23–7.17 (m, 3H), 7.06–6.99 (m, 2H), 4.30 (q, *J* = 6.6 Hz, 1H), 3.51–3.43 (m, 2H), 2.88 (d, *J* = 9.1 Hz, 1H), 2.44 (d, *J* = 11.7 Hz, 1H), 2.32 (s, 3H), 2.07–1.94 (m, 3H), 1.76 (d, *J* = 11.8 Hz, 1H), 1.34 (d, *J* = 6.6 Hz, 3H), 1.14 (t, *J* = 11.1 Hz, 1H), 0.28 (d, *J* = 13.2 Hz, 1H); ^13^C NMR (100 MHz, CDCl_3_) δ 143.8, 139.3, 136.3, 129.6, 129.1, 128.3, 128.2, 127.1, 126.6, 124.0, 123.0, 115.7, 64.5, 63.4, 51.4, 50.1, 45.7, 38.6, 28.9, 21.5, 17.6; HRMS (ESI+) calcd for C_27_H_31_N_2_O_2_S^+^ [M + H]^+^: 447.2101. Found: 447.2108.

## 4. Conclusions

A new method for the synthesis of 3,3-dialkyl indolenines has been developed utilizing the Lewis acid promoted alkylation of indoles with trichloroacetimidates. This method is differentiated from past methods in that it does not depend on transition metal mediated alkylation or the use of strong base, instead a Lewis acid and a trichloroacetimidate leaving group are utilized to perform the alkylation. Notably even electron poor indoles undergo the dialkylation, which are difficult substrates for other alkylation reactions. The indolenines generated from this reaction provide ready access to spirocyclic structures which are useful platforms for the development of three dimensional architectures that may interact with more complex biological receptors of interest to the medicinal chemistry community.

## Figures and Tables

**Table 1:**
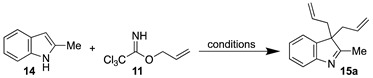
Optimization of the diallylation reaction.

Entry	Equiv Imidate 11	Equiv TMSOTf	Temp. (°C)	Reaction Time	Yield (%)
1	2.2	0	84	24 h	0
2	2.2	0.2	rt	3 h	27
3	2.2	0.2	rt	6 h	20
4	2.2	0.2	84	3 h	41
5	3.0	0.2	rt	3 h	31
6	2.5	0.5	rt	3 h	39
7	2.5	1.0	rt	3 h	61
8	2.5	1.5	rt	3 h	59
9	2.5	1.0	84	3 h	59

Conditions: 1,2-Dichloroethane (DCE), TMSOTf, rt or reflux.

**Table 2:**
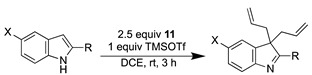
C3-Dialkylation of functionalized indoles with allyl imidate.

Entry	Indole	Product	Yield (%)
1	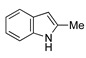 **14a**	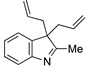 **15a**	61
2	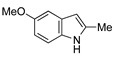 **14b**	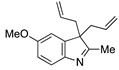 **15b**	61
3	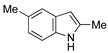 **14c**	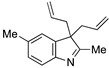 **15c**	41
4	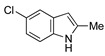 **14d**	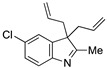 **15d**	45
5	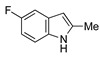 **14e**	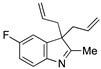 **15e**	68
6	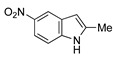 **10**	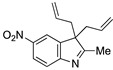 **13**	70
7	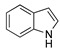 **14f**	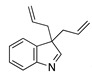 **15f**	0 ^a^
8	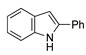 **14g**	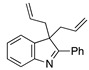 **15g**	34
9	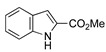 **14h**	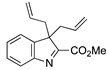 **15h**	0 ^b^

^a^ A complex mixture resulted. ^b^ Starting material was recovered.

**Table 3:**
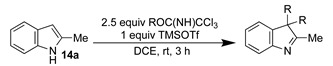
Direct C3-dialkylation of 2-methyl indole **14a** with trichloroacetimidates.

Entry	Imidate	Product	Yield (%)
1	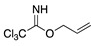 **11**	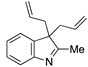 **15a**	61 (59 ^a^)
2	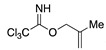 **16**	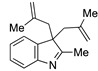 **15i**	40 (46 ^a^)
3	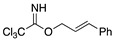 **17**	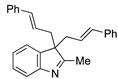 **15j**	12 (20 ^a^)
4	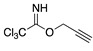 **18**	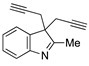 **15k**	trace (24 ^a^)
5	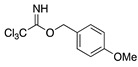 **19**	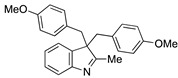 **15l**	0 ^b^
6	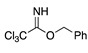 **20**	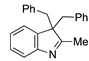 **15m**	30 (38 ^a^)
7	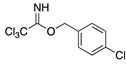 **21**	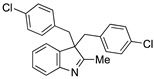 **15n**	trace (52 ^a^)
8	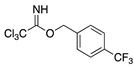 **22**	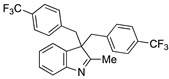 **15o**	trace (45 ^a^)
9	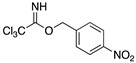 **23**	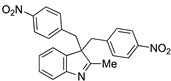 **15p**	trace (63 ^a^)

^a^ Yield when the reaction was performed at reflux. ^b^ A complex mixture resulted.

## Supplementary Materials

Copies of ^1^H and ^13^C spectra are available at https://www.mdpi.com/1420-3049/24/22/4143/s1.
