# Supplementary material for: Dialkylation of Indoles with Trichloroacetimidates to Access 3,3-Disubstituted Indolenines
Source: Molecules. 2019 Nov 15;24(22):4143. doi: 10.3390/molecules24224143 (PMC6891773; doi:10.3390/molecules24224143)
Supplement: Supplementary file 1 [file molecules-24-04143-s001.pdf]

# Supporting Information

# Dialkylation of Indoles with Trichloroacetimidates to Access 3,3-Disubstituted Indolenines

*Tamie Suzuki, Nilamber A. Mate, Arijit A. Adhikari and John D. Chisholm**

Department of Chemistry, 1-014 Center for Science and Technology

Syracuse University, Syracuse, NY 13244

*jdchisho@syr.edu*

**Contents**

Table of Contents S1

$^{1}$H and $^{13}$C NMR Spectra S2-S20

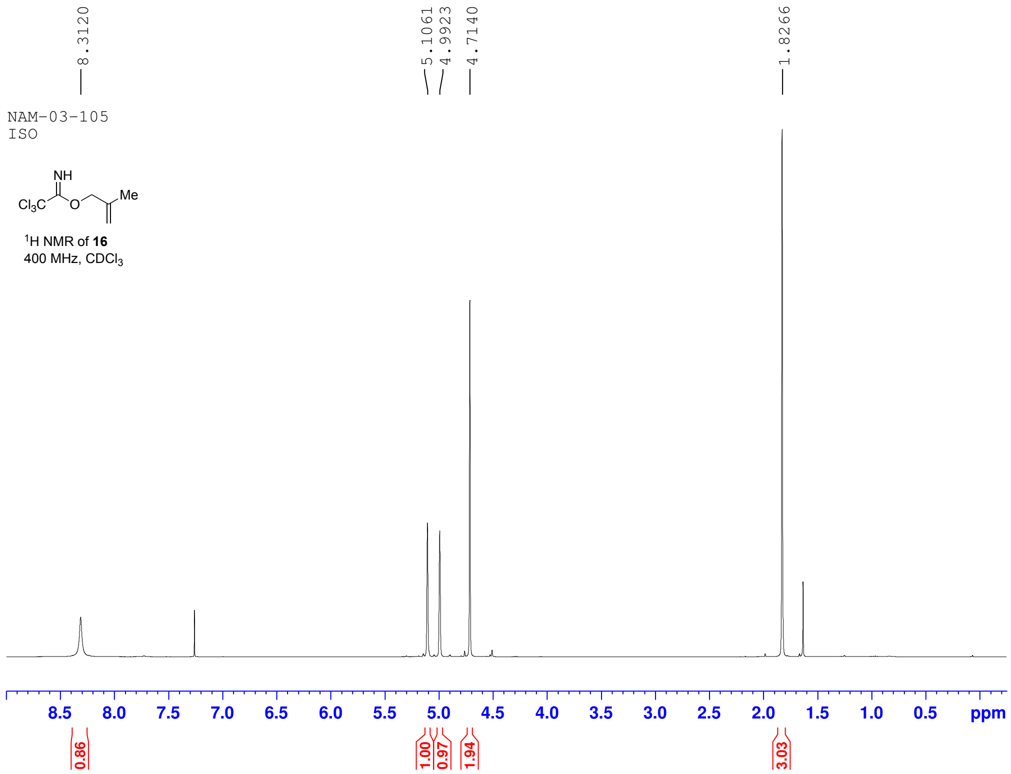
8.3120
5.1061
4.9923
4.7140
1.8266
NAM-03-105
ISO
NH
Cl3C
O
Me
1H NMR of 16
400 MHz, CDCl3
8.5 8.0 7.5 7.0 6.5 6.0 5.5 5.0 4.5 4.0 3.5 3.0 2.5 2.0 1.5 1.0 0.5 ppm
0.86
1.00
0.97
1.94
3.03

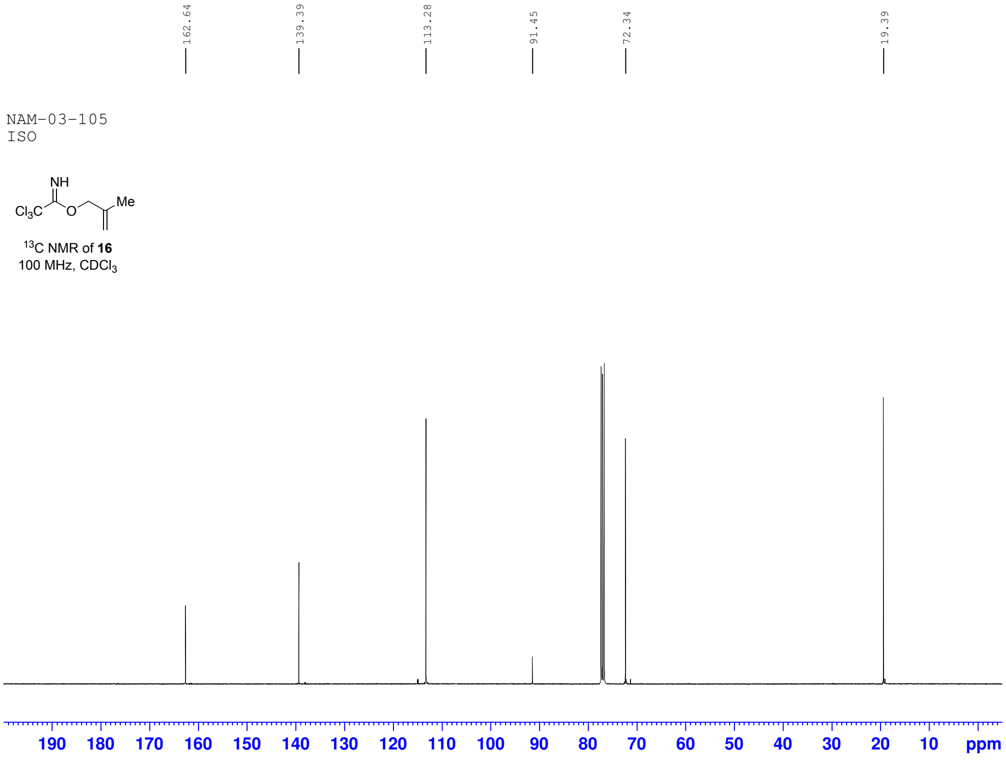
162.64
139.39
113.28
91.45
72.34
19.39
NAM-03-105
ISO
NH
Cl3C
O
Me
13C NMR of 16
100 MHz, CDCl3
190 180 170 160 150 140 130 120 110 100 90 80 70 60 50 40 30 20 10 ppm

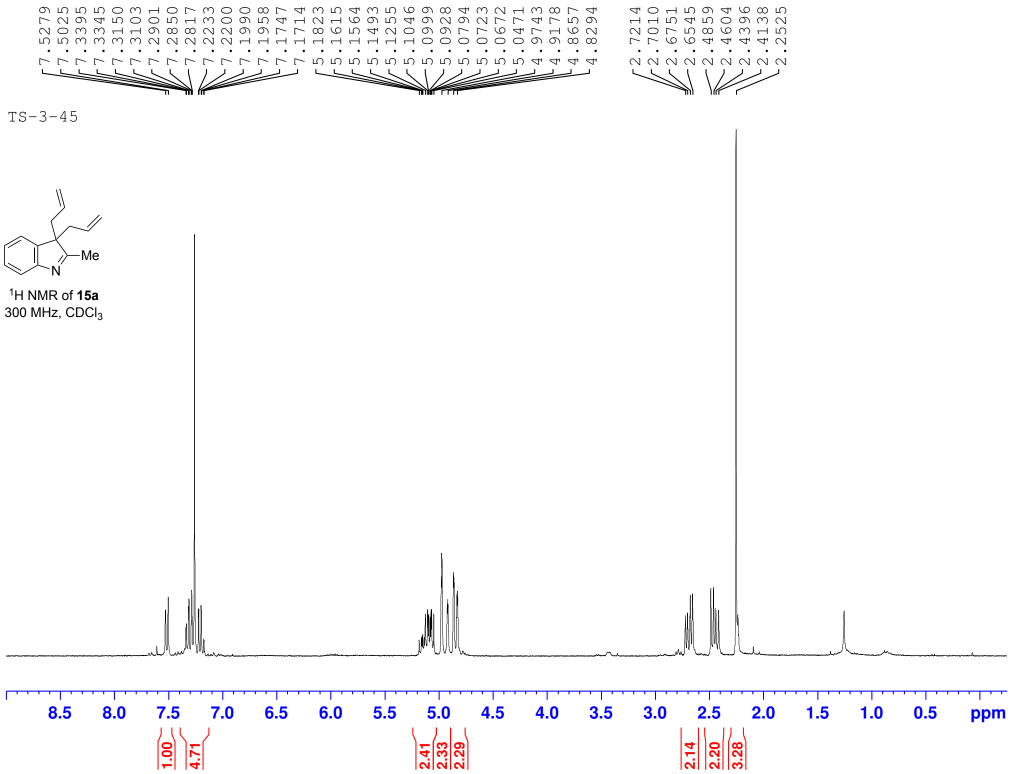
7.5279
7.5025
7.3395
7.3345
7.3150
7.3103
7.2901
7.2850
7.2817
7.2233
7.2200
7.1990
7.1958
7.1747
7.1714
5.1823
5.1615
5.1564
5.1493
5.1255
5.1046
5.0999
5.0928
5.0794
5.0723
5.0672
5.0471
4.9743
4.9178
4.8657
4.8294
2.7214
2.7010
2.6751
2.6545
2.4859
2.4604
2.4396
2.4138
2.2525
TS-3-45
Me
N
$^{1}$H NMR of **15a**
300 MHz, $CDCl_3$
8.5 8.0 7.5 7.0 6.5 6.0 5.5 5.0 4.5 4.0 3.5 3.0 2.5 2.0 1.5 1.0 0.5 ppm
1.00
4.71
2.41
2.33
2.29
2.14
2.20
3.28

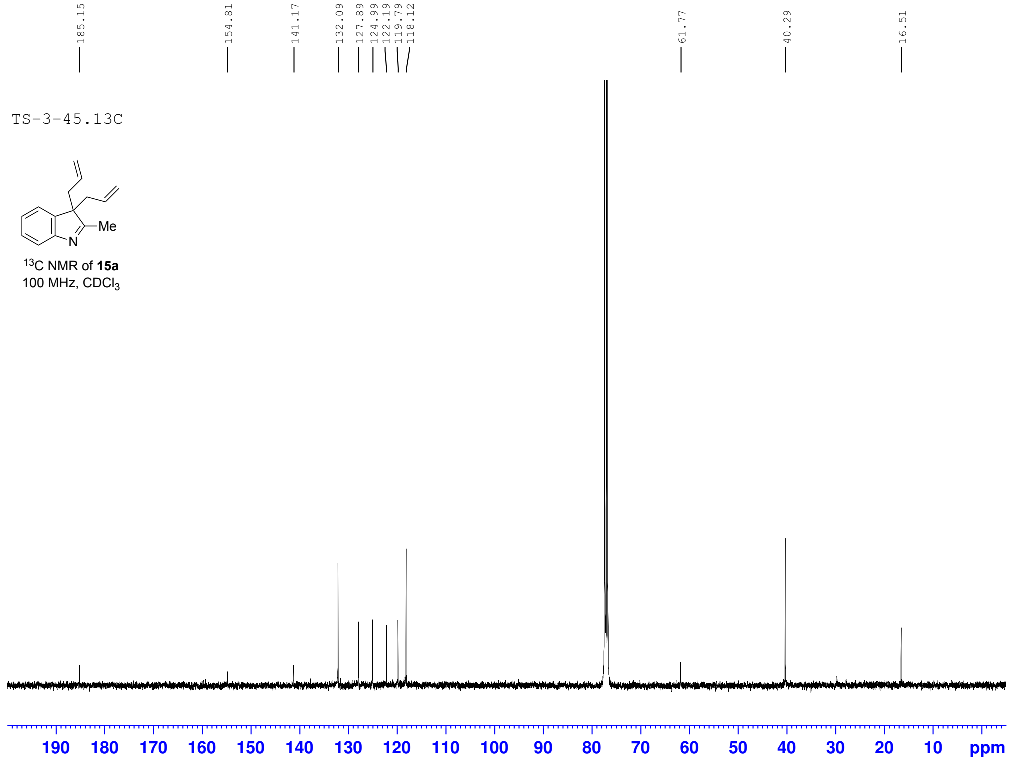
185.15
154.81
141.17
132.09
127.89
124.99
122.19
119.79
118.12
61.77
40.29
16.51
TS-3-45.13C
Me
N
$^{13}$C NMR of **15a**
100 MHz, $CDCl_3$
190 180 170 160 150 140 130 120 110 100 90 80 70 60 50 40 30 20 10 ppm

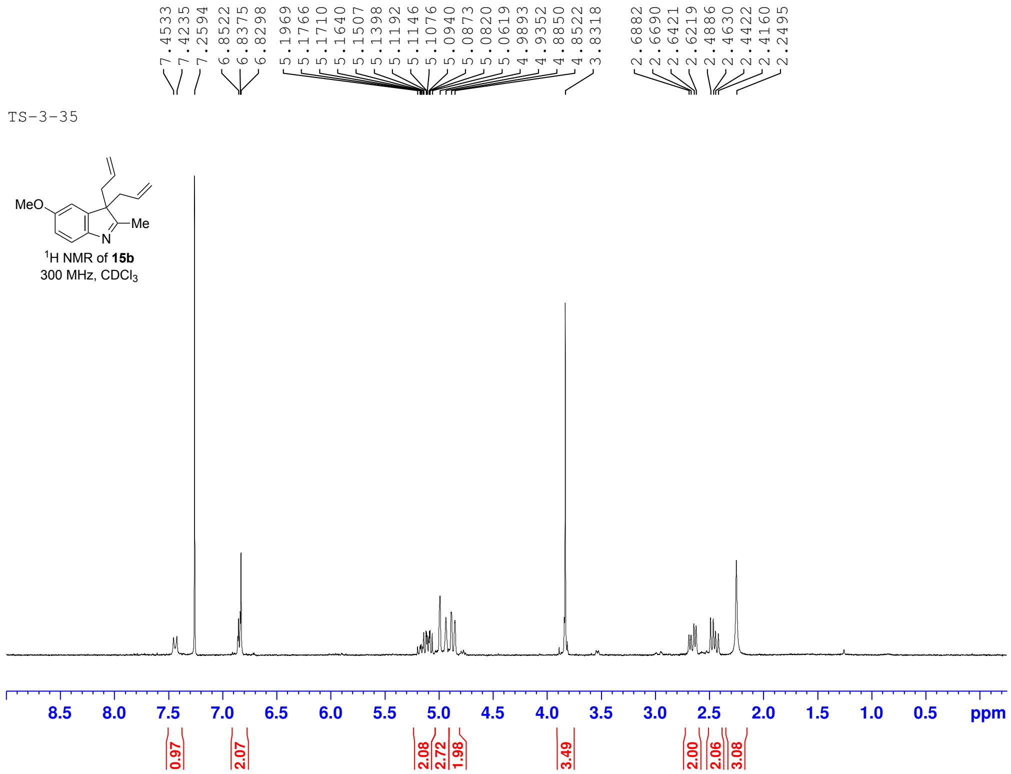
7.4533
7.4235
7.2594
6.8522
6.8375
6.8298
5.1969
5.1766
5.1710
5.1640
5.1507
5.1398
5.1192
5.1146
5.1076
5.0940
5.0873
5.0820
5.0619
4.9893
4.9352
4.8850
4.8522
3.8318
2.6882
2.6690
2.6421
2.6219
2.4886
2.4630
2.4422
2.4160
2.2495
TS-3-35
MeO
N
Me
$^{1}$H NMR of **15b**
300 MHz, $CDCl_3$
8.5 8.0 7.5 7.0 6.5 6.0 5.5 5.0 4.5 4.0 3.5 3.0 2.5 2.0 1.5 1.0 0.5 ppm
0.97
2.07
2.08
2.72
1.98
3.49
2.00
2.06
3.08

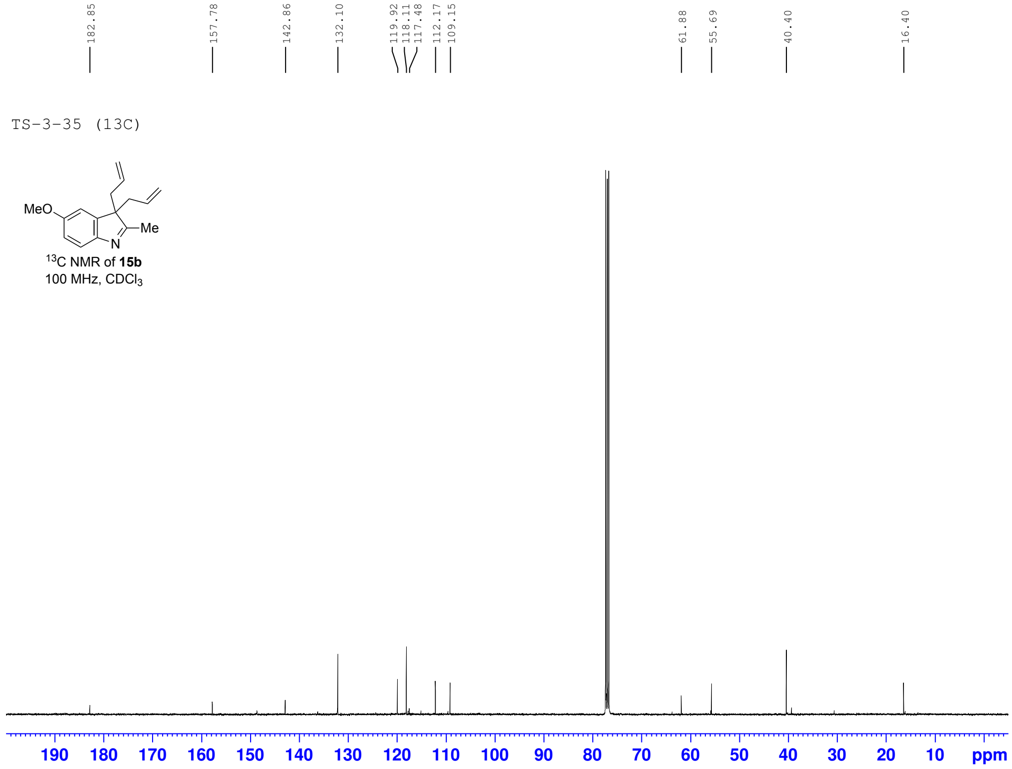
182.85
157.78
142.86
132.10
119.92
118.11
117.48
112.17
109.15
61.88
55.69
40.40
16.40
TS-3-35 (13C)
MeO
N
Me
$^{13}$C NMR of **15b**
100 MHz, $CDCl_3$
190 180 170 160 150 140 130 120 110 100 90 80 70 60 50 40 30 20 10 ppm

TS-3-34 (1H)

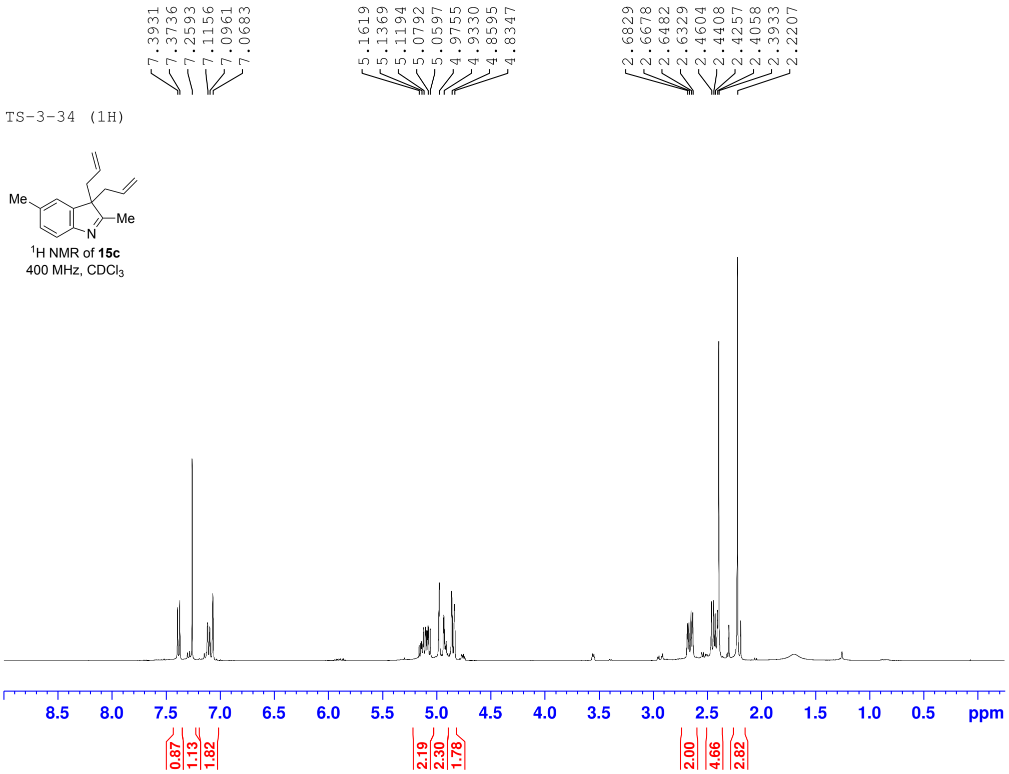


$^1$H NMR of **15c**
400 MHz, $CDCl_3$

TS-3-34 (13C)

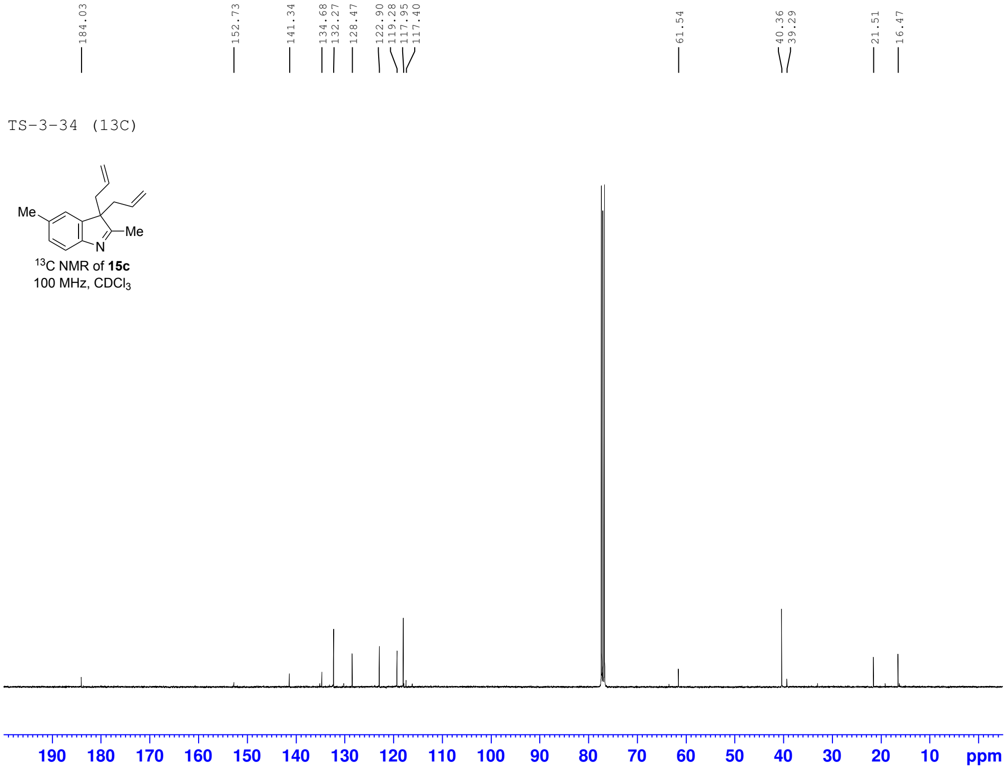


$^{13}$C NMR of **15c**
100 MHz, $CDCl_3$

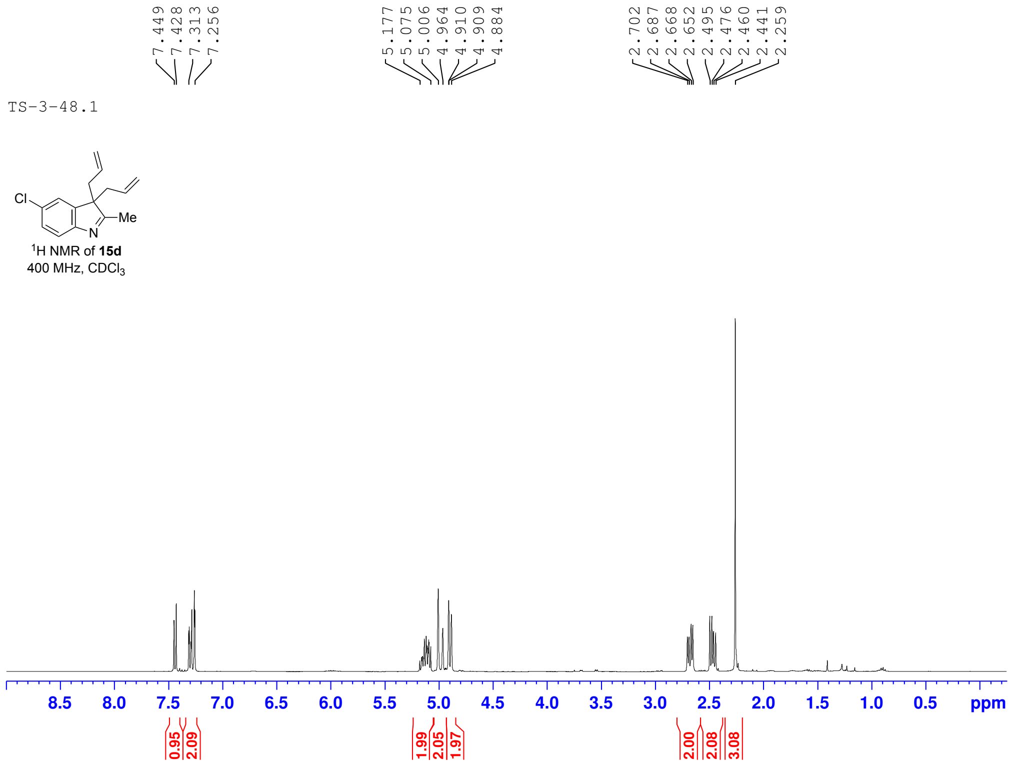
7.449
7.428
7.313
7.256
5.177
5.075
5.006
4.964
4.910
4.909
4.884
2.702
2.687
2.668
2.652
2.495
2.476
2.460
2.441
2.259
TS-3-48.1
Cl
Me
N
$^1$H NMR of **15d**
400 MHz, $CDCl_3$
8.5 8.0 7.5 7.0 6.5 6.0 5.5 5.0 4.5 4.0 3.5 3.0 2.5 2.0 1.5 1.0 0.5 ppm
0.95
2.09
1.99
2.05
1.97
2.00
2.08
3.08

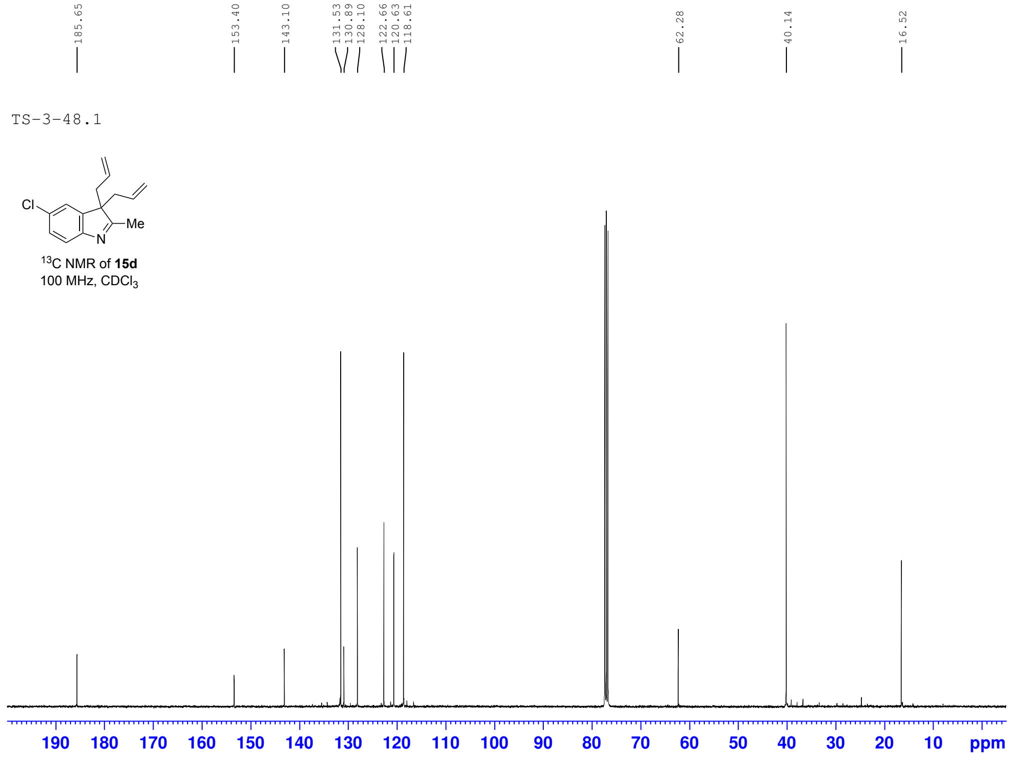
185.65
153.40
143.10
131.53
130.89
128.10
122.66
120.63
118.61
62.28
40.14
16.52
TS-3-48.1
Cl
Me
N
$^{13}$C NMR of **15d**
100 MHz, $CDCl_3$
190 180 170 160 150 140 130 120 110 100 90 80 70 60 50 40 30 20 10 ppm

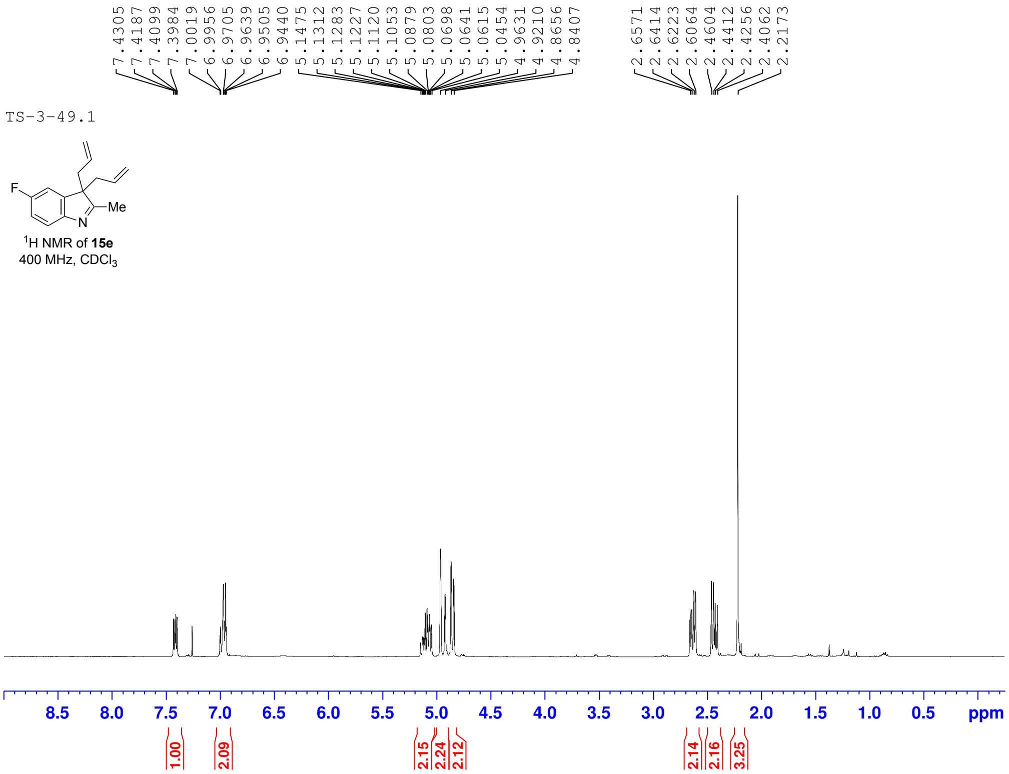


$^{1}H$ NMR of **15e**
400 MHz, $CDCl_3$

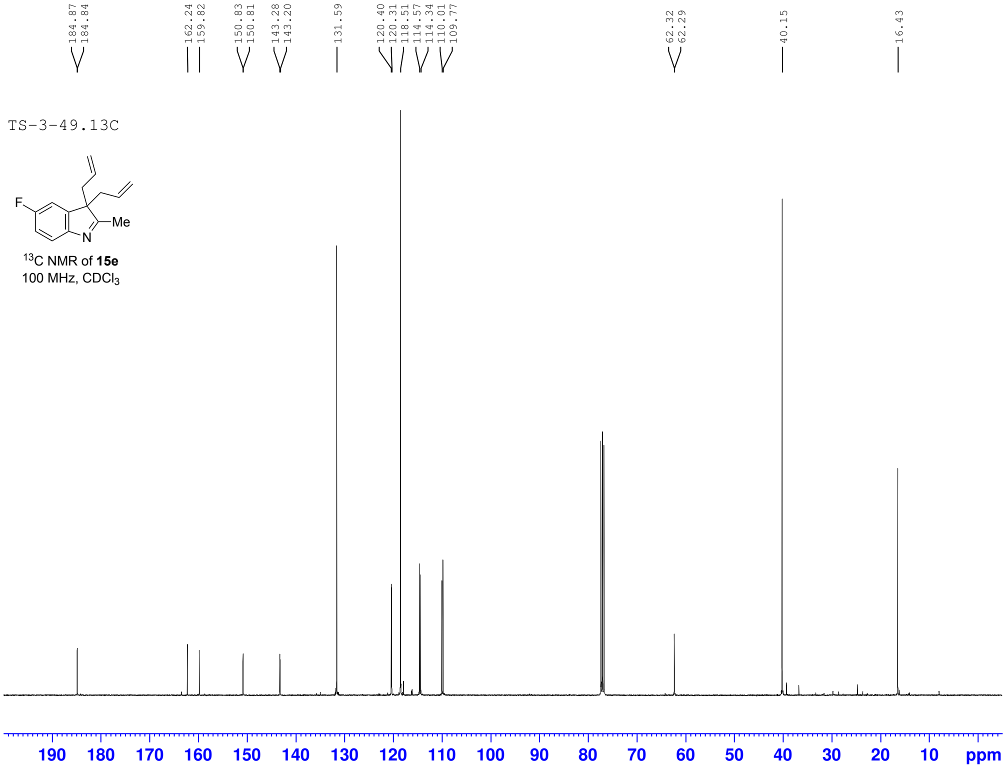


$^{13}C$ NMR of **15e**
100 MHz, $CDCl_3$

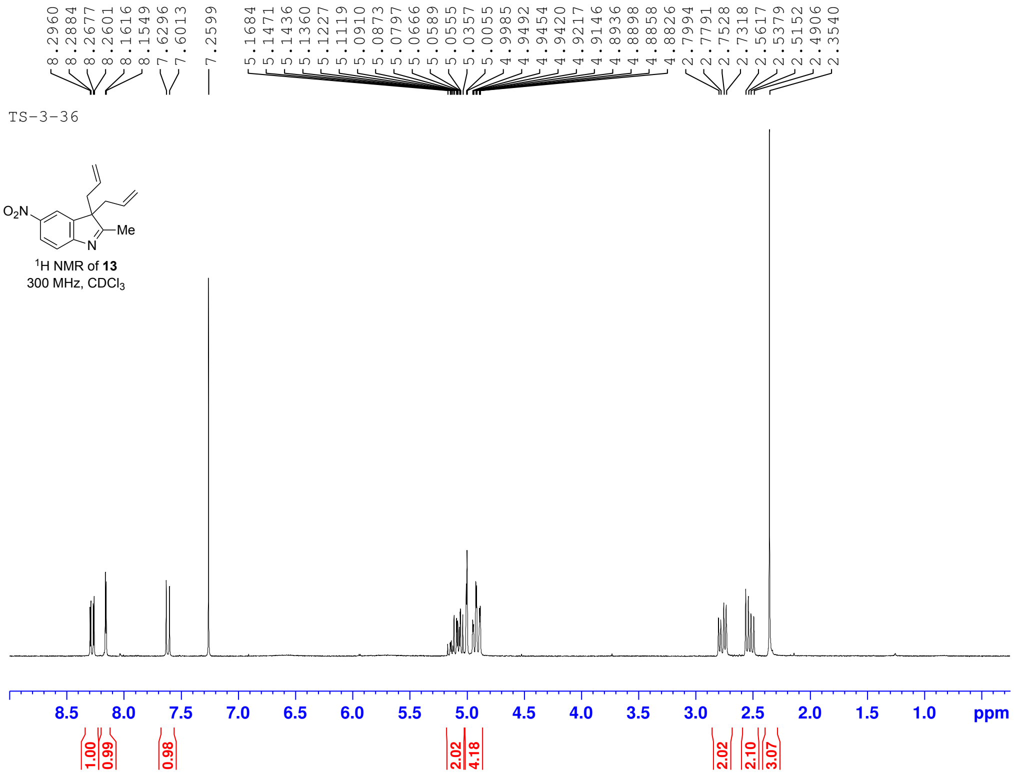
TS-3-36
$O_2N$
Me
N
$^1$H NMR of **13**
300 MHz, $CDCl_3$
ppm

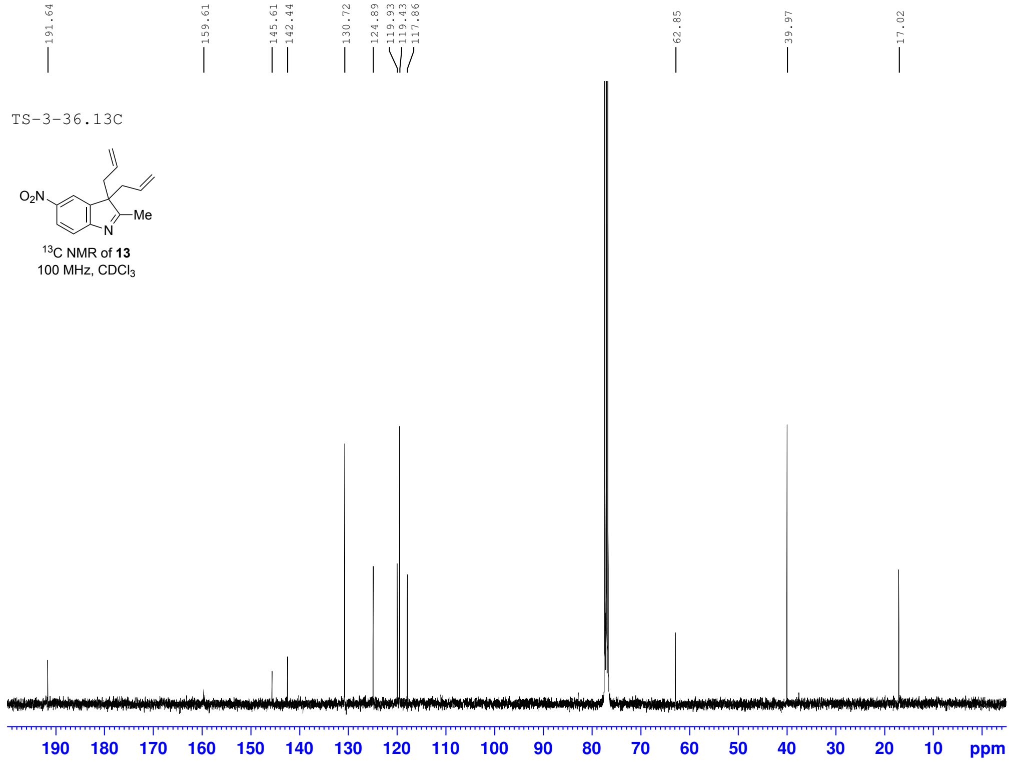
TS-3-36.13C
$O_2N$
Me
N
$^{13}$C NMR of **13**
100 MHz, $CDCl_3$
191.64
159.61
145.61
142.44
130.72
124.89
119.93
119.43
117.86
62.85
39.97
17.02
ppm

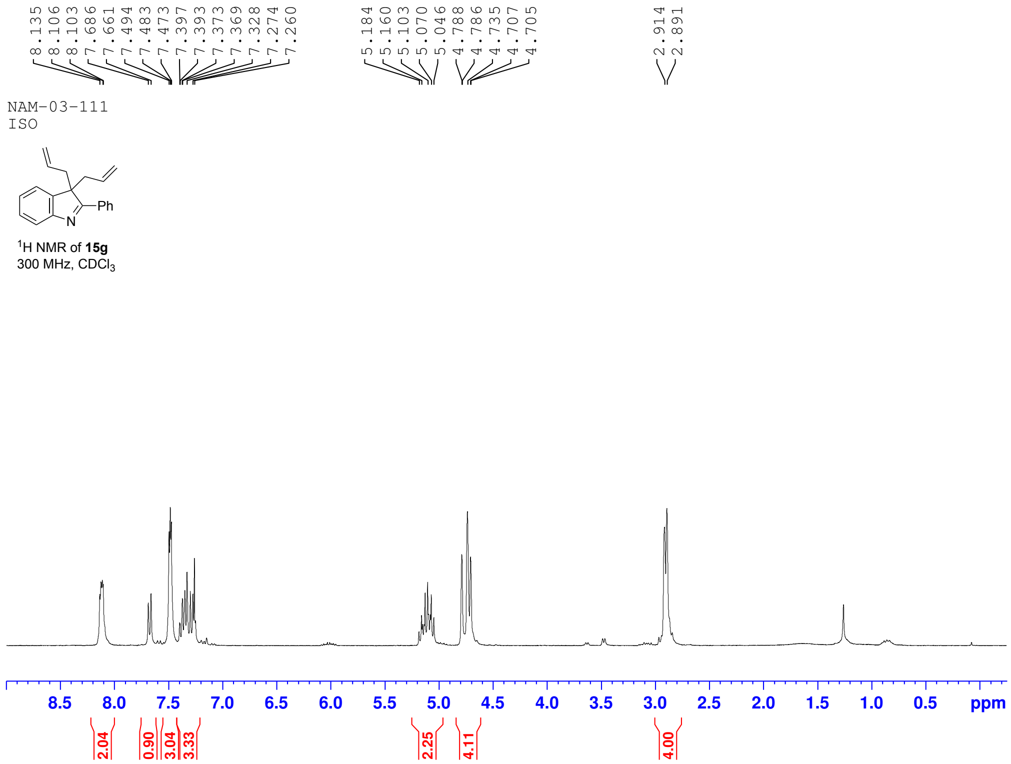
8.135
8.106
8.103
7.686
7.661
7.494
7.483
7.473
7.397
7.393
7.373
7.369
7.328
7.274
7.260
5.184
5.160
5.103
5.070
5.046
4.788
4.786
4.735
4.707
4.705
2.914
2.891
NAM-03-111
ISO
Ph
N
$^{1}$H NMR of **15g**
300 MHz, $CDCl_3$
2.04
0.90
3.04
3.33
2.25
4.11
4.00
ppm

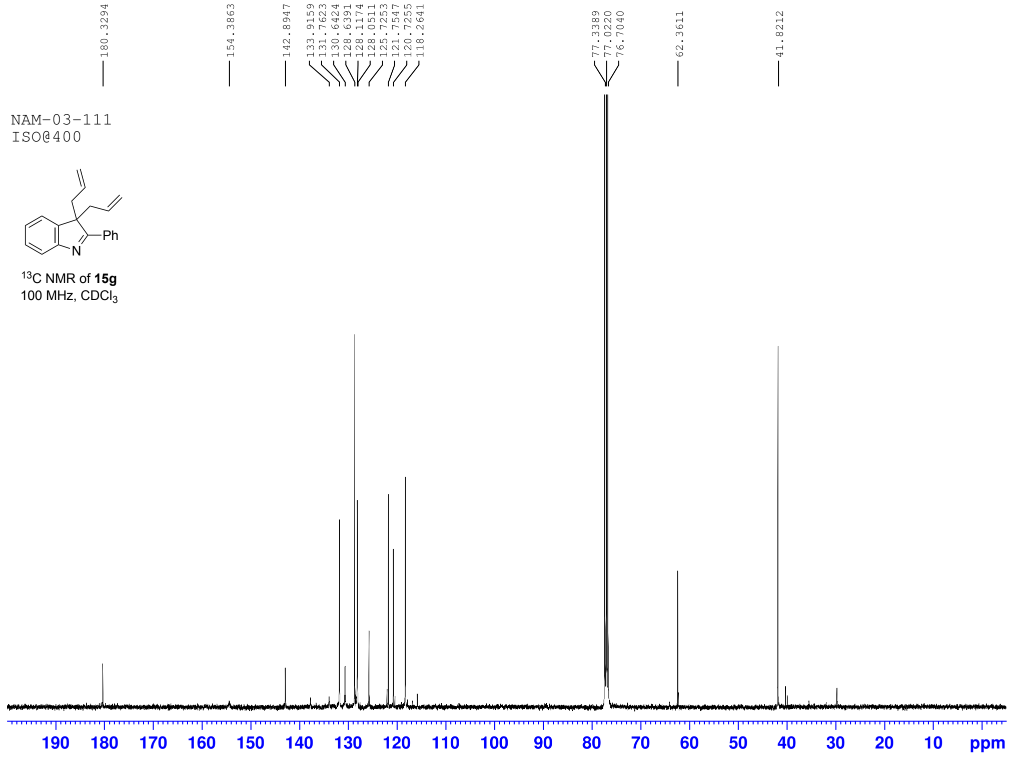
180.3294
154.3863
142.8947
133.9159
131.7623
130.6424
128.6391
128.1174
128.0511
125.7253
121.7547
120.7255
118.2641
77.3389
77.0220
76.7040
62.3611
41.8212
NAM-03-111
ISO@400
Ph
N
$^{13}$C NMR of **15g**
100 MHz, $CDCl_3$
ppm

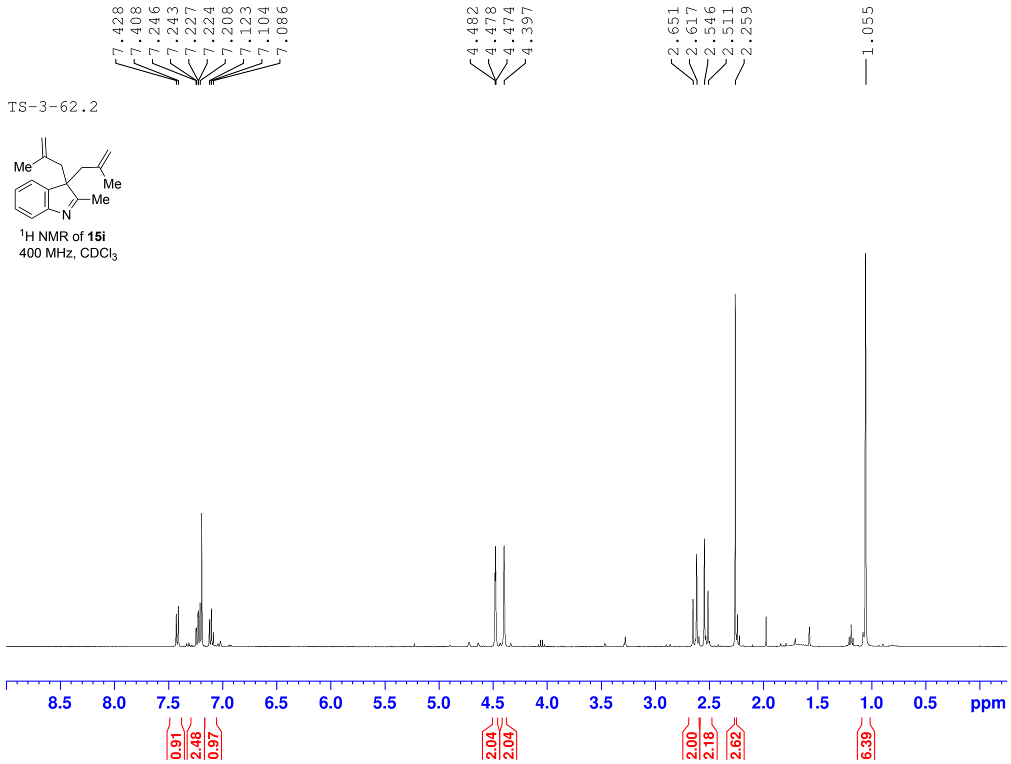
7.428
7.408
7.246
7.243
7.227
7.224
7.208
7.123
7.104
7.086
4.482
4.478
4.474
4.397
2.651
2.617
2.546
2.511
2.259
1.055
TS-3-62.2
Me
Me
Me
N
$^1$H NMR of **15i**
400 MHz, $CDCl_3$
8.5 8.0 7.5 7.0 6.5 6.0 5.5 5.0 4.5 4.0 3.5 3.0 2.5 2.0 1.5 1.0 0.5 ppm
0.91 2.48 0.97
2.04 2.04
2.00 2.18 2.62
6.39

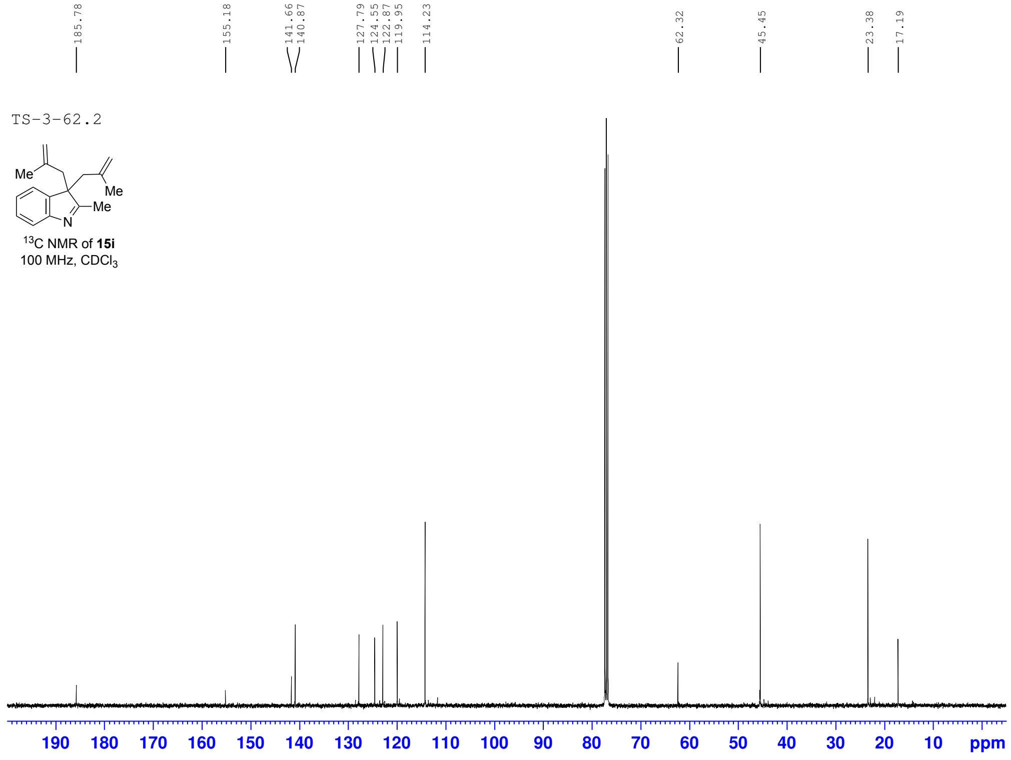
185.78
155.18
141.66
140.87
127.79
124.55
122.87
119.95
114.23
62.32
45.45
23.38
17.19
TS-3-62.2
Me
Me
Me
N
$^{13}$C NMR of **15i**
100 MHz, $CDCl_3$
190 180 170 160 150 140 130 120 110 100 90 80 70 60 50 40 30 20 10 ppm

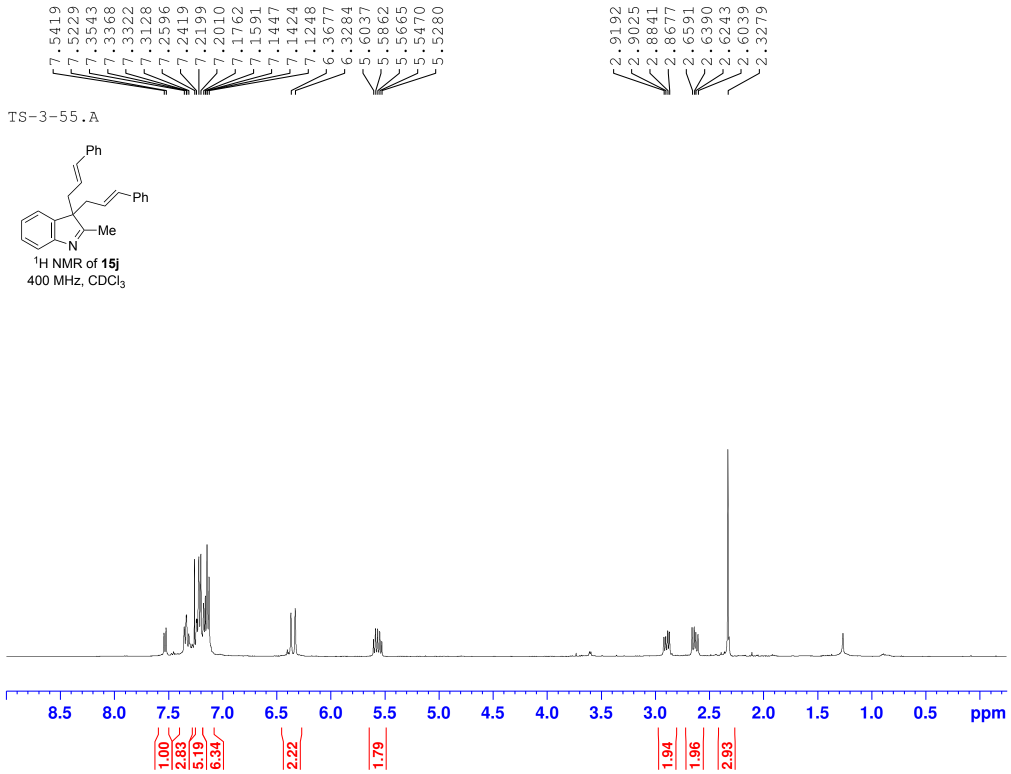
7.5419
7.5229
7.3543
7.3368
7.3322
7.3128
7.2596
7.2419
7.2199
7.2010
7.1762
7.1591
7.1447
7.1424
7.1248
6.3677
6.3284
5.6037
5.5862
5.5665
5.5470
5.5280
2.9192
2.9025
2.8841
2.8677
2.6591
2.6390
2.6243
2.6039
2.3279
TS-3-55.A
Ph
Ph
Me
N
$^1$H NMR of **15j**
400 MHz, $CDCl_3$
8.5 8.0 7.5 7.0 6.5 6.0 5.5 5.0 4.5 4.0 3.5 3.0 2.5 2.0 1.5 1.0 0.5 ppm
1.00
2.83
5.19
6.34
2.22
1.79
1.94
1.96
2.93

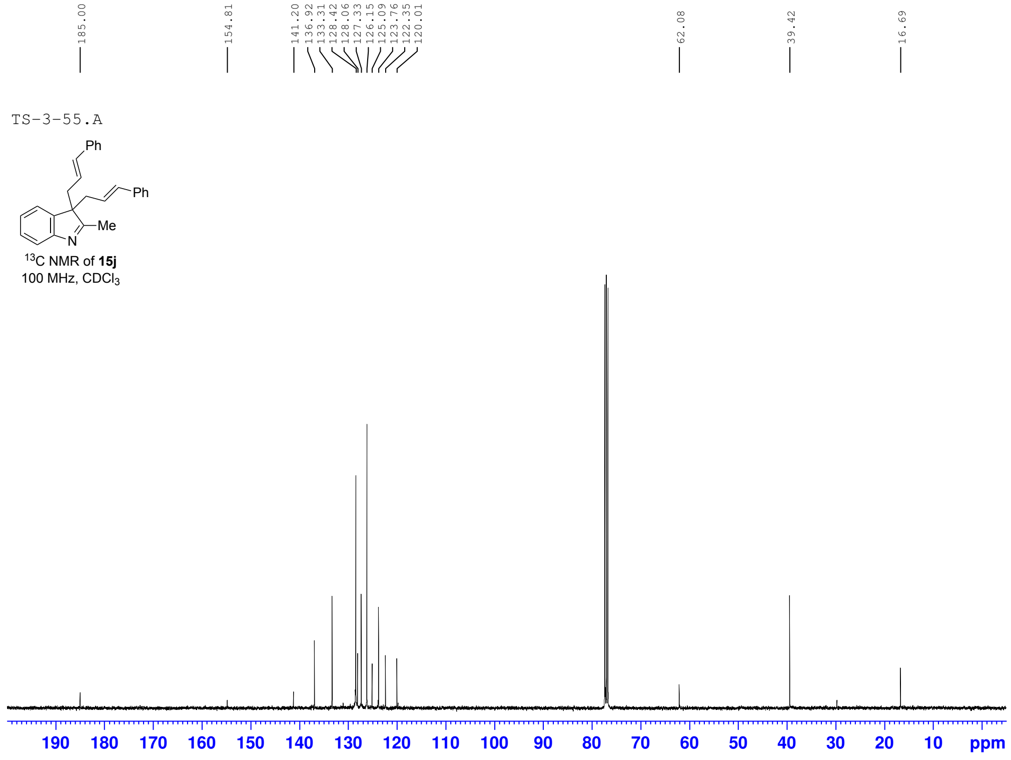
185.00
154.81
141.20
136.92
133.31
128.42
128.06
127.33
126.15
125.09
123.76
122.35
120.01
62.08
39.42
16.69
TS-3-55.A
Ph
Ph
Me
N
$^{13}$C NMR of **15j**
100 MHz, $CDCl_3$
190 180 170 160 150 140 130 120 110 100 90 80 70 60 50 40 30 20 10 ppm

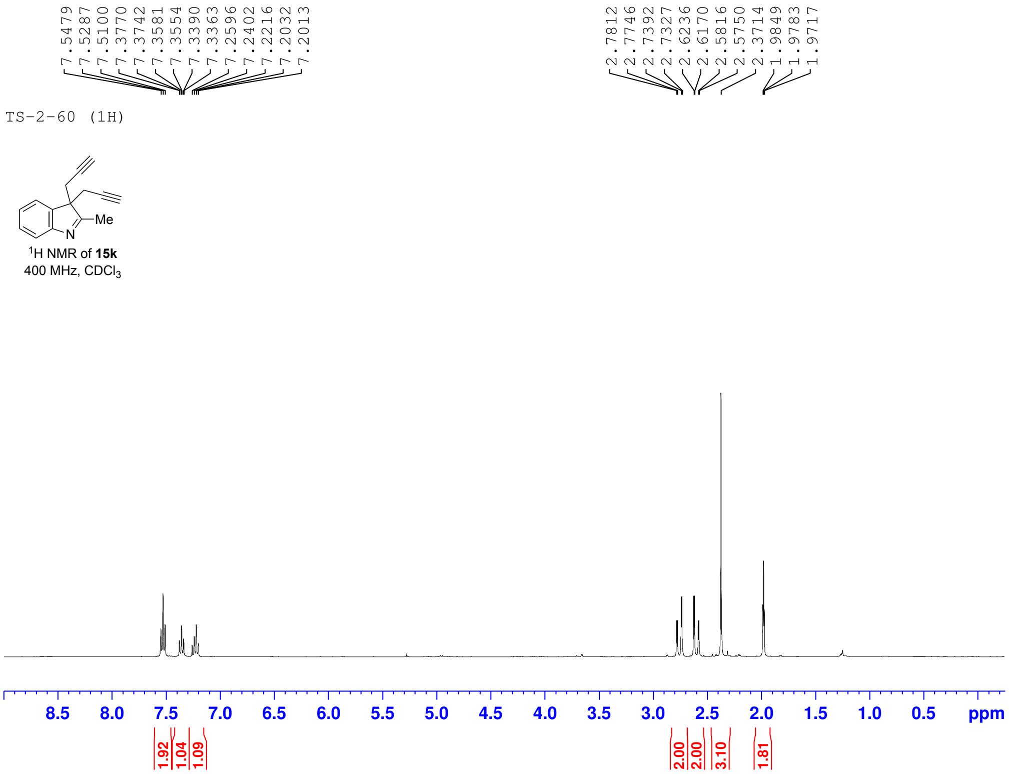
7.5479
7.5287
7.5100
7.3770
7.3742
7.3581
7.3554
7.3390
7.3363
7.2596
7.2402
7.2216
7.2032
7.2013
2.7812
2.7746
2.7392
2.7327
2.6236
2.6170
2.5816
2.5750
2.3714
1.9849
1.9783
1.9717
TS-2-60 (1H)
Me
N
1H NMR of 15k
400 MHz, CDCl3
8.5 8.0 7.5 7.0 6.5 6.0 5.5 5.0 4.5 4.0 3.5 3.0 2.5 2.0 1.5 1.0 0.5 ppm
1.92
1.04
1.09
2.00
2.00
3.10
1.81

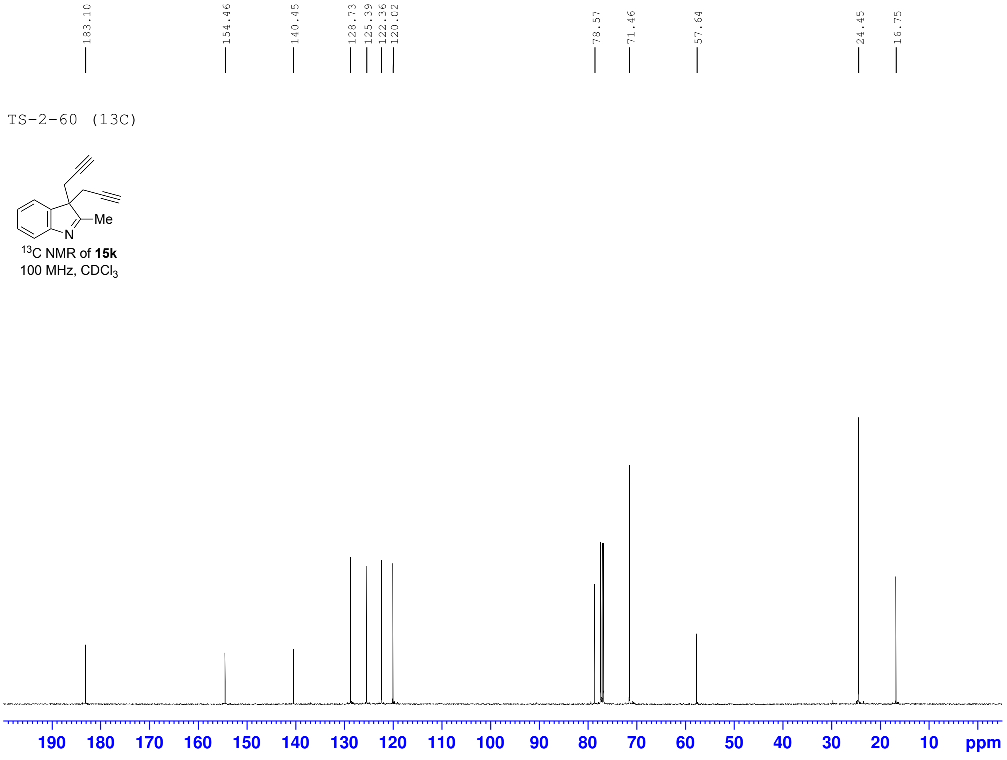
183.10
154.46
140.45
128.73
125.39
122.36
120.02
78.57
71.46
57.64
24.45
16.75
TS-2-60 (13C)
Me
N
13C NMR of 15k
100 MHz, CDCl3
190 180 170 160 150 140 130 120 110 100 90 80 70 60 50 40 30 20 10 ppm

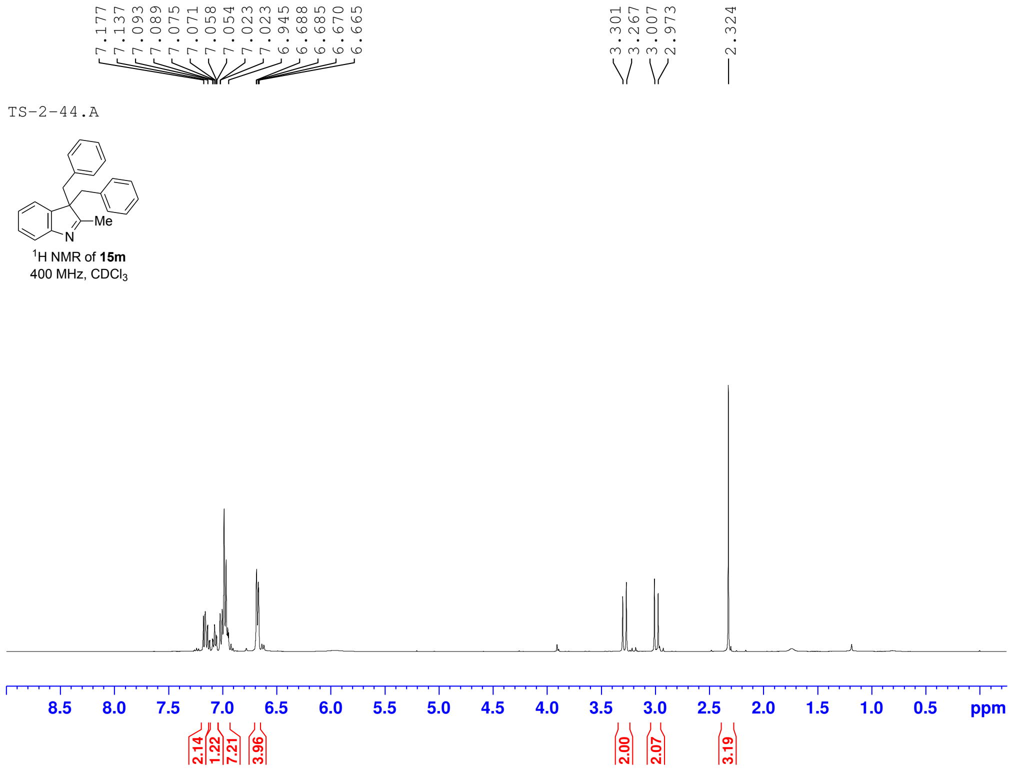
7.177
7.137
7.093
7.089
7.075
7.071
7.058
7.054
7.023
7.023
6.945
6.688
6.685
6.670
6.665
3.301
3.267
3.007
2.973
2.324
TS-2-44.A
Me
N
$^{1}$H NMR of **15m**
400 MHz, CDCl3
8.5
8.0
7.5
7.0
6.5
6.0
5.5
5.0
4.5
4.0
3.5
3.0
2.5
2.0
1.5
1.0
0.5
ppm
2.14
1.22
7.21
3.96
2.00
2.07
3.19

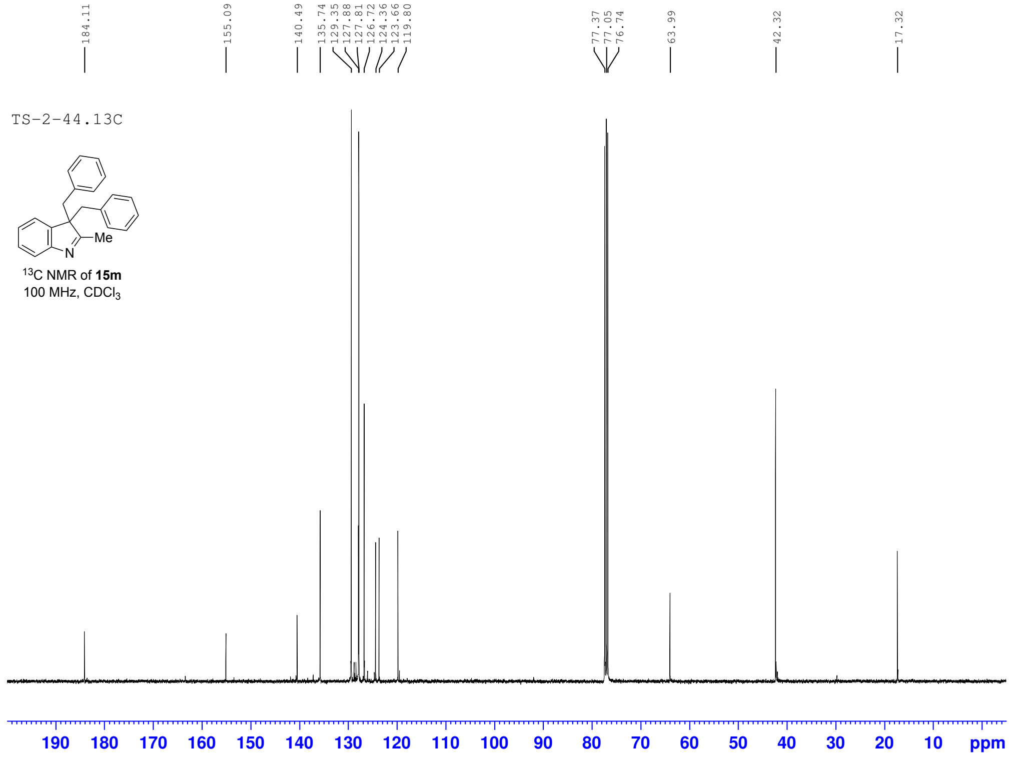
184.11
155.09
140.49
135.74
129.35
127.88
127.81
126.72
124.36
123.66
119.80
77.37
77.05
76.74
63.99
42.32
17.32
TS-2-44.13C
Me
N
$^{13}$C NMR of **15m**
100 MHz, CDCl3
190
180
170
160
150
140
130
120
110
100
90
80
70
60
50
40
30
20
10
ppm

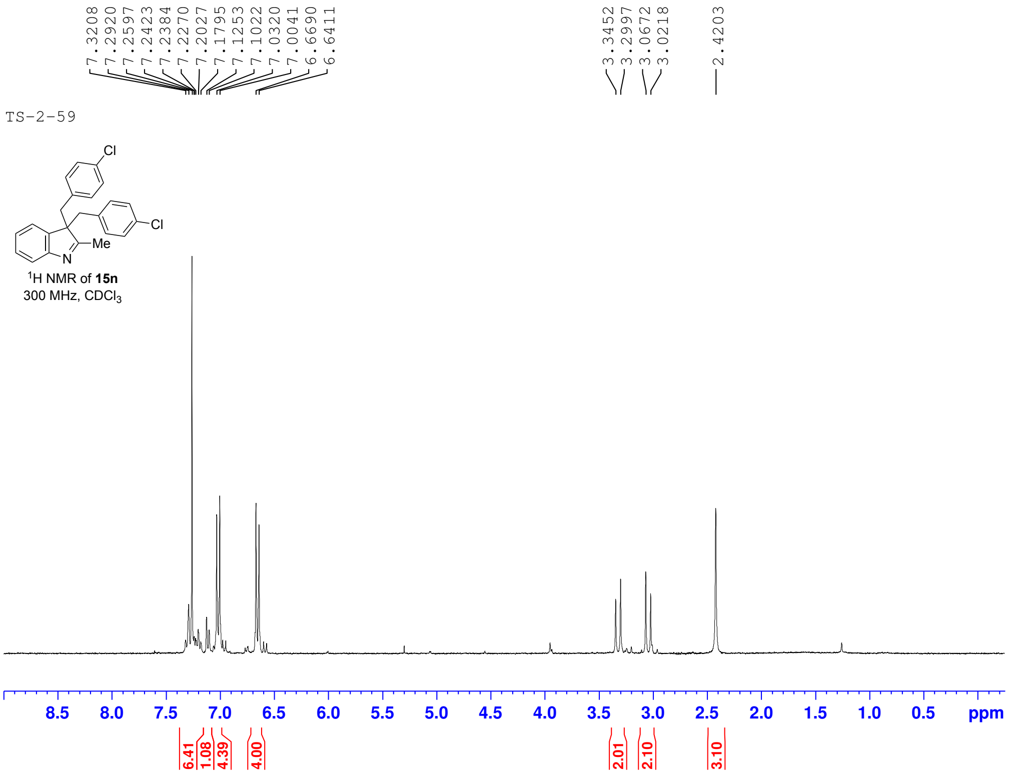
7.3208
7.2920
7.2597
7.2423
7.2384
7.2270
7.2027
7.1795
7.1253
7.1022
7.0320
7.0041
6.6690
6.6411
3.3452
3.2997
3.0672
3.0218
2.4203
TS-2-59
Cl
Cl
Me
N
1H NMR of 15n
300 MHz, CDCl3
8.5 8.0 7.5 7.0 6.5 6.0 5.5 5.0 4.5 4.0 3.5 3.0 2.5 2.0 1.5 1.0 0.5 ppm
6.41
1.08
4.39
4.00
2.01
2.10
3.10

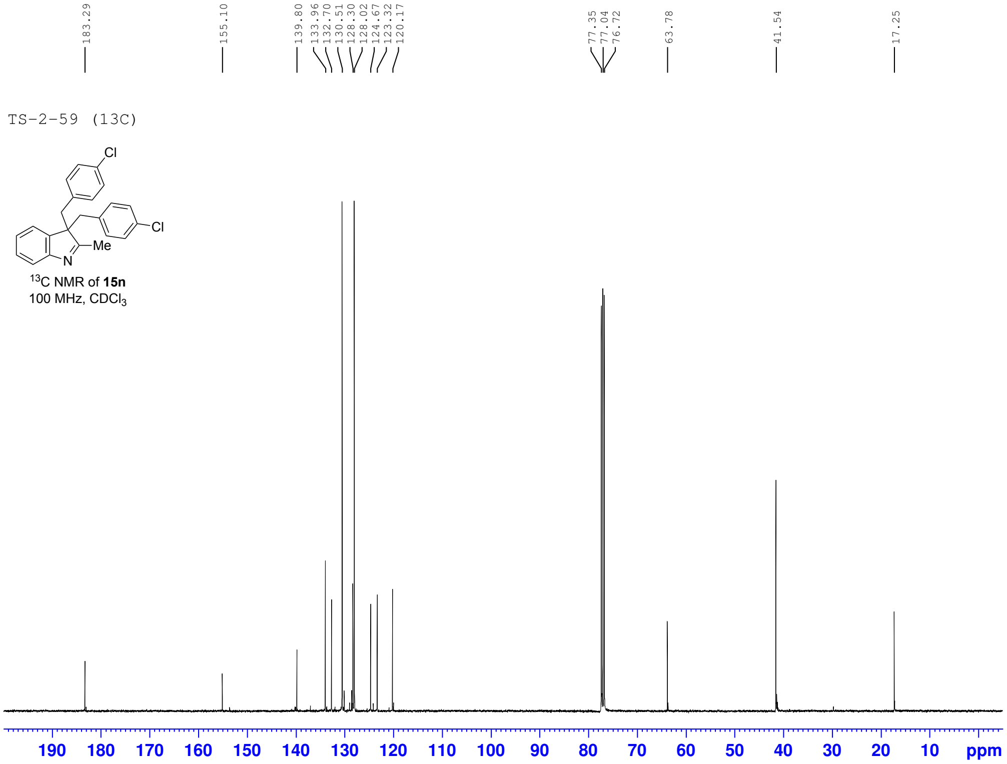
183.29
155.10
139.80
133.96
132.70
130.51
128.10
128.02
124.67
123.32
120.17
77.35
77.04
76.72
63.78
41.54
17.25
TS-2-59 (13C)
Cl
Cl
Me
N
13C NMR of 15n
100 MHz, CDCl3
190 180 170 160 150 140 130 120 110 100 90 80 70 60 50 40 30 20 10 ppm

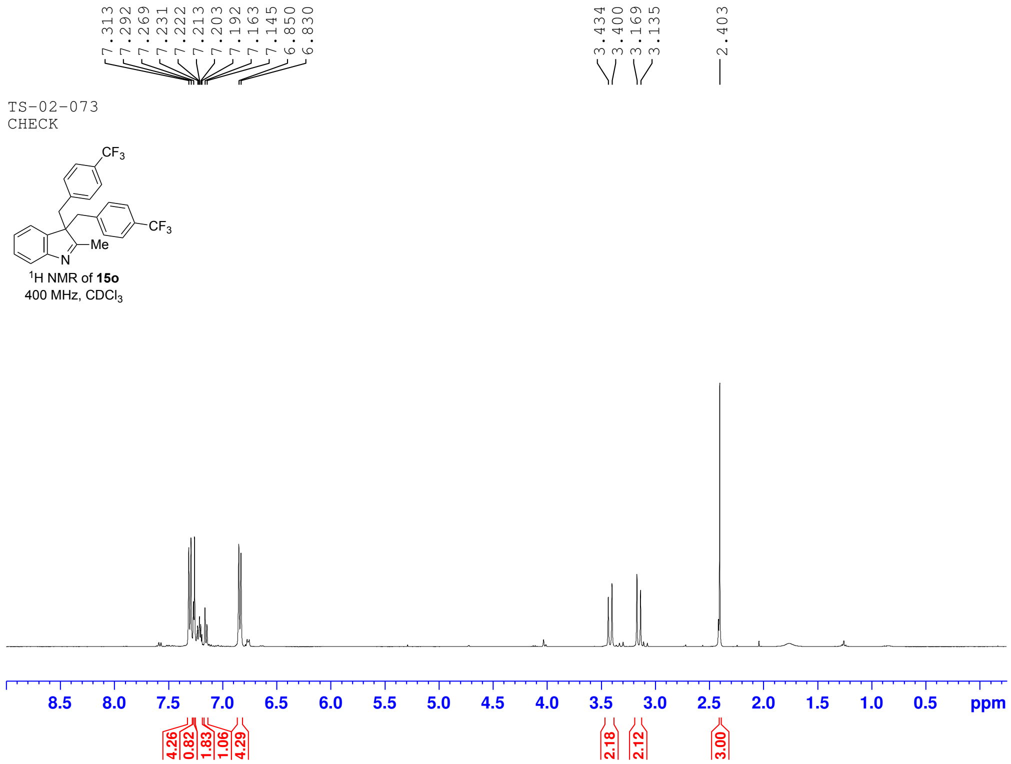
7.313
7.292
7.269
7.231
7.222
7.213
7.203
7.192
7.163
7.145
6.850
6.830
3.434
3.400
3.169
3.135
2.403
TS-02-073
CHECK
CF3
CF3
Me
N
1H NMR of 15o
400 MHz, CDCl3
8.5 8.0 7.5 7.0 6.5 6.0 5.5 5.0 4.5 4.0 3.5 3.0 2.5 2.0 1.5 1.0 0.5 ppm
4.26
0.82
1.83
1.06
4.29
2.18
2.12
3.00

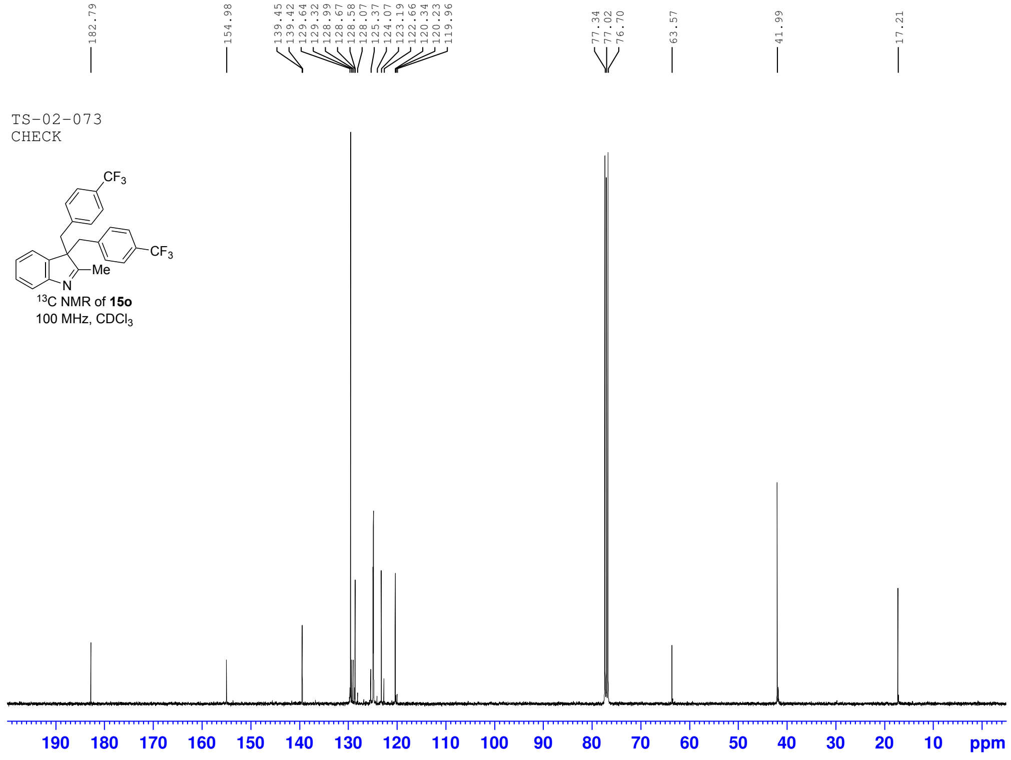
182.79
154.98
139.45
139.42
129.64
129.32
128.99
128.67
128.58
128.07
125.37
124.07
123.19
122.66
120.34
120.23
119.96
77.34
77.02
76.70
63.57
41.99
17.21
TS-02-073
CHECK
CF3
CF3
Me
N
13C NMR of 15o
100 MHz, CDCl3
190 180 170 160 150 140 130 120 110 100 90 80 70 60 50 40 30 20 10 ppm

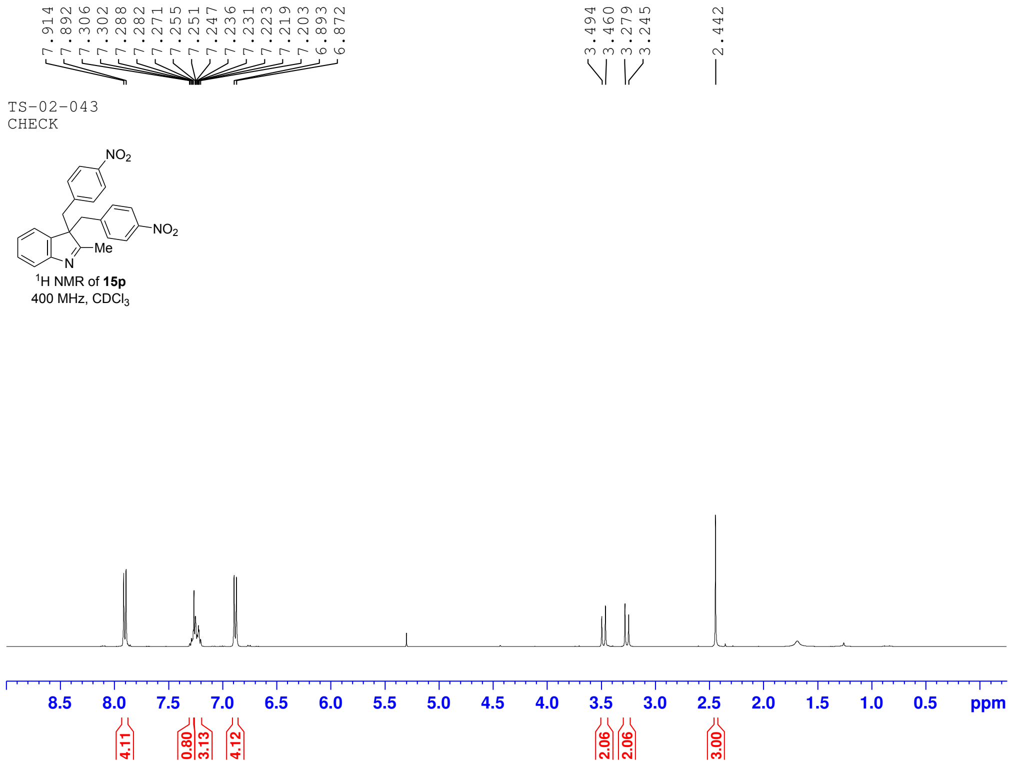

7.914
7.892
7.306
7.302
7.288
7.282
7.271
7.255
7.251
7.247
7.236
7.231
7.223
7.219
7.203
6.893
6.872
3.494
3.460
3.279
3.245
2.442
TS-02-043
CHECK
NO2
NO2
Me
N
1H NMR of 15p
400 MHz, CDCl3
8.5
8.0
7.5
7.0
6.5
6.0
5.5
5.0
4.5
4.0
3.5
3.0
2.5
2.0
1.5
1.0
0.5
ppm
4.11
0.80
3.13
4.12
2.06
2.06
3.00


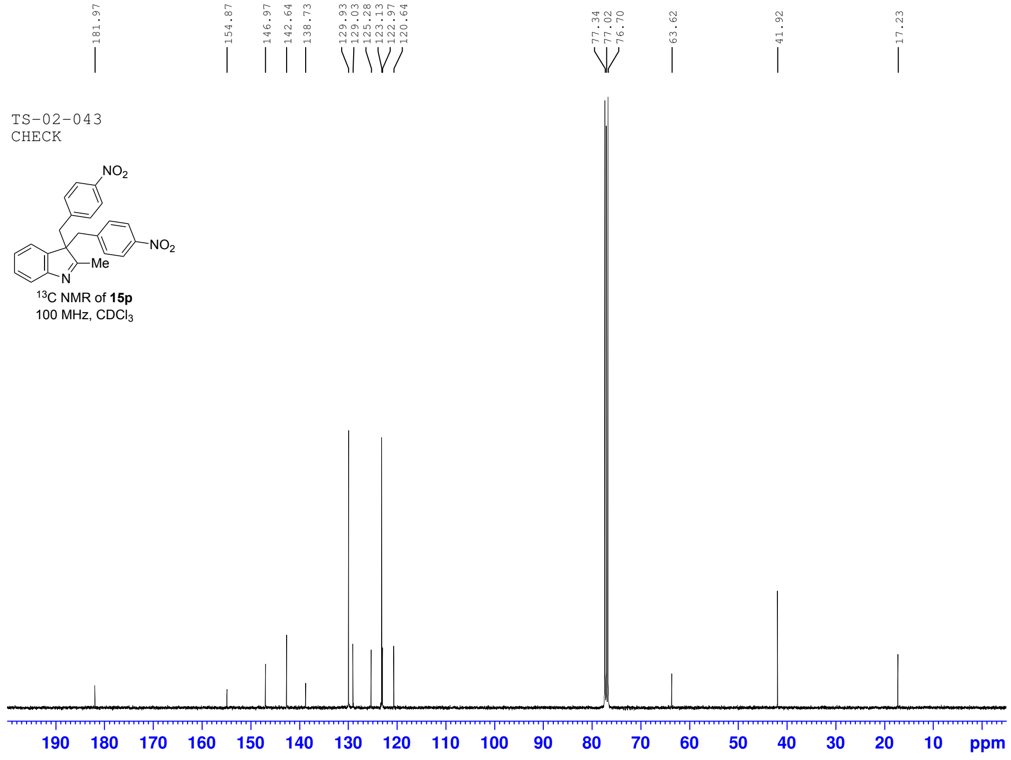

181.97
154.87
146.97
142.64
138.73
129.93
129.03
125.28
123.13
122.97
120.64
77.34
77.02
76.70
63.62
41.92
17.23
TS-02-043
CHECK
NO2
NO2
Me
N
13C NMR of 15p
100 MHz, CDCl3
190
180
170
160
150
140
130
120
110
100
90
80
70
60
50
40
30
20
10
ppm

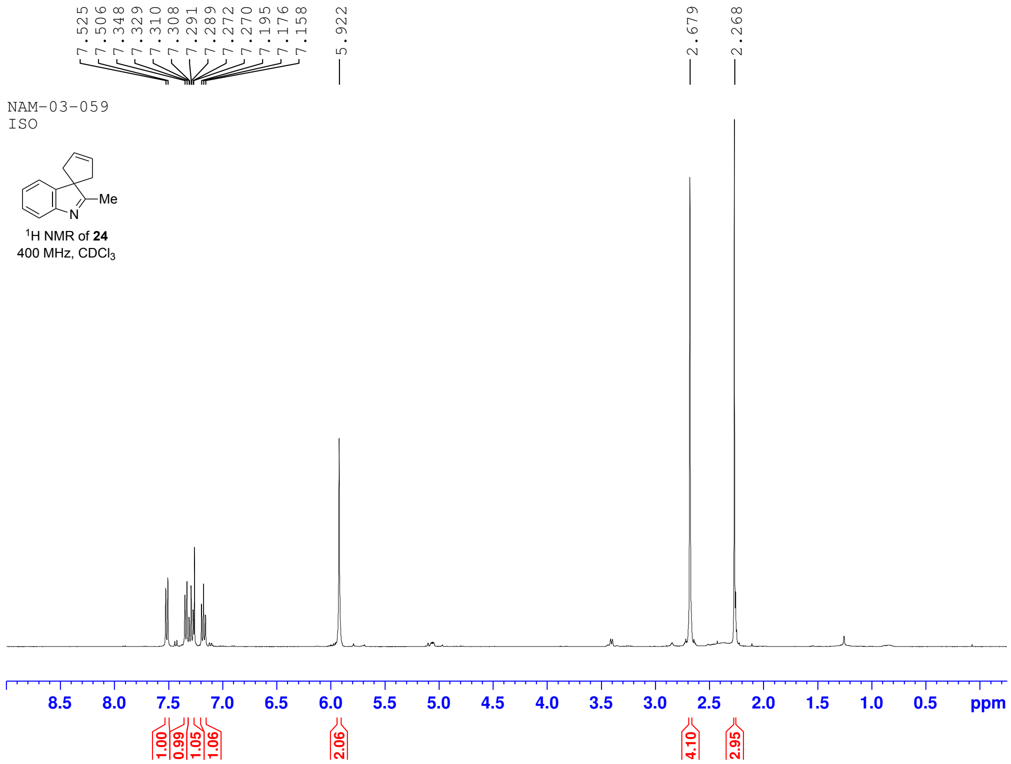
7.525
7.506
7.348
7.329
7.310
7.308
7.291
7.289
7.272
7.270
7.195
7.176
7.158
5.922
2.679
2.268
NAM-03-059
ISO
Me
N
$^{1}$H NMR of **24**
400 MHz, $CDCl_3$
8.5
8.0
7.5
7.0
6.5
6.0
5.5
5.0
4.5
4.0
3.5
3.0
2.5
2.0
1.5
1.0
0.5
ppm
1.00
0.99
1.05
1.06
2.06
4.10
2.95

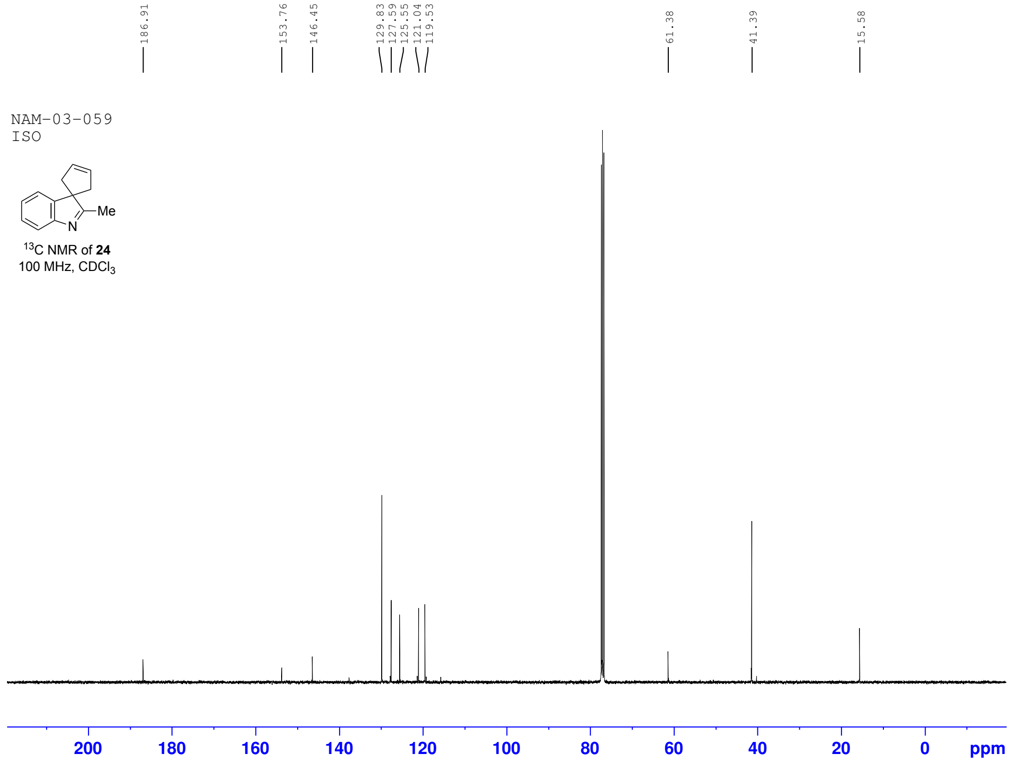
186.91
153.76
146.45
129.83
127.59
125.55
121.04
119.53
61.38
41.39
15.58
NAM-03-059
ISO
Me
N
$^{13}$C NMR of **24**
100 MHz, $CDCl_3$
200
180
160
140
120
100
80
60
40
20
0
ppm

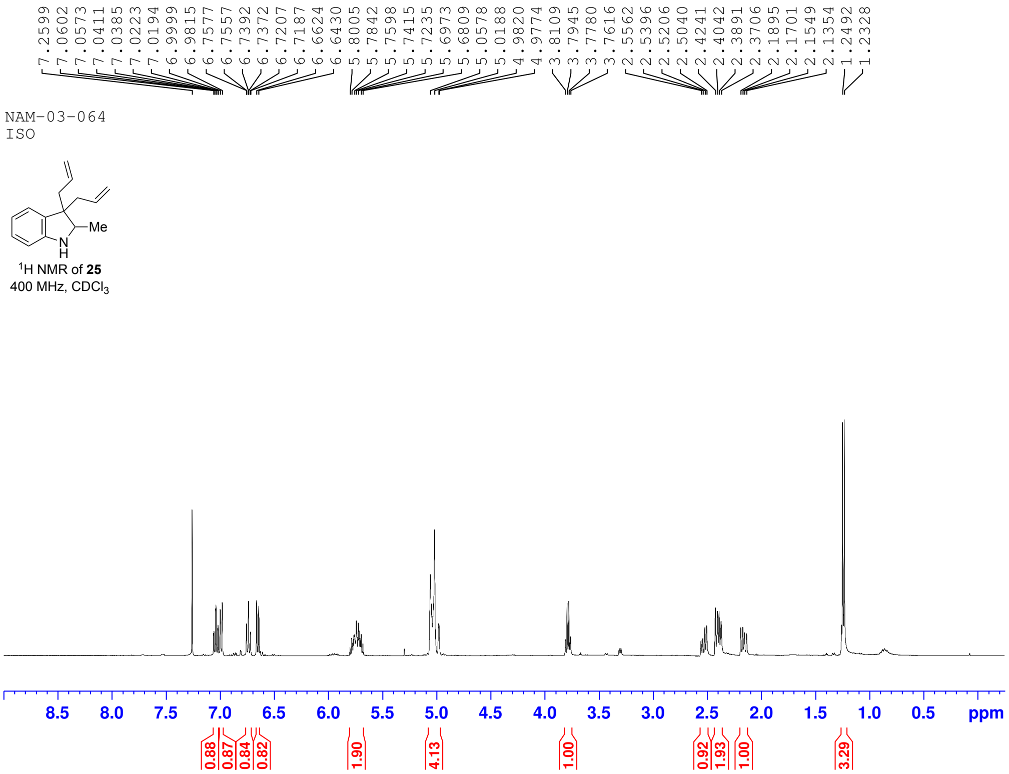


$^{1}H$ NMR of **25**
400 MHz, $CDCl_3$

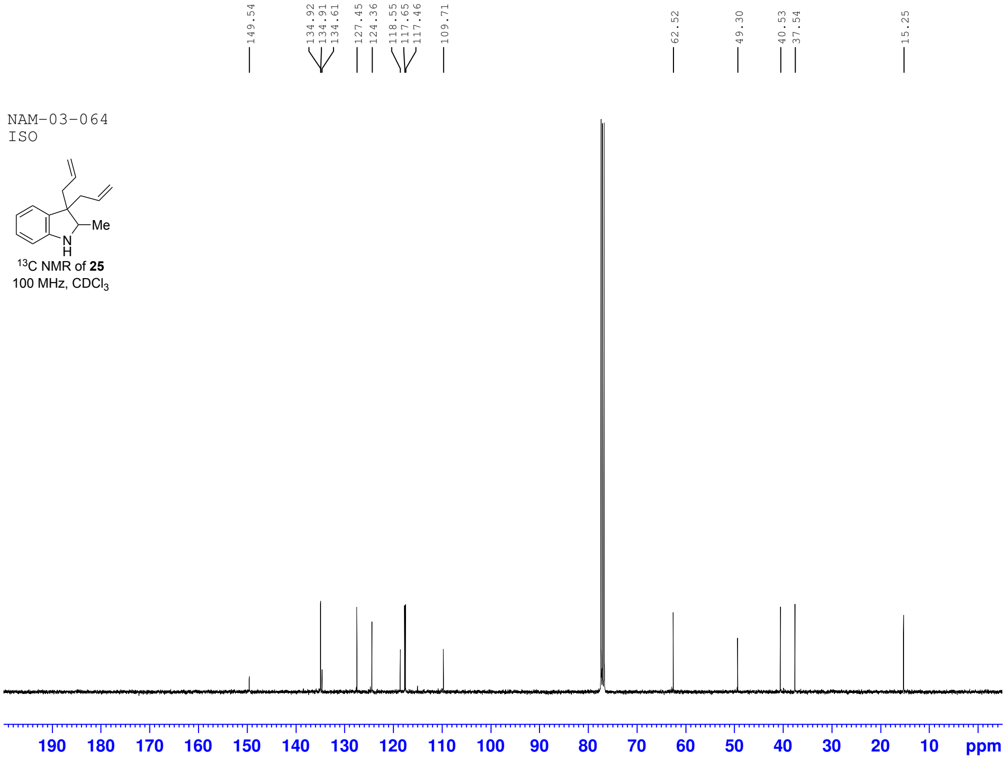


$^{13}C$ NMR of **25**
100 MHz, $CDCl_3$

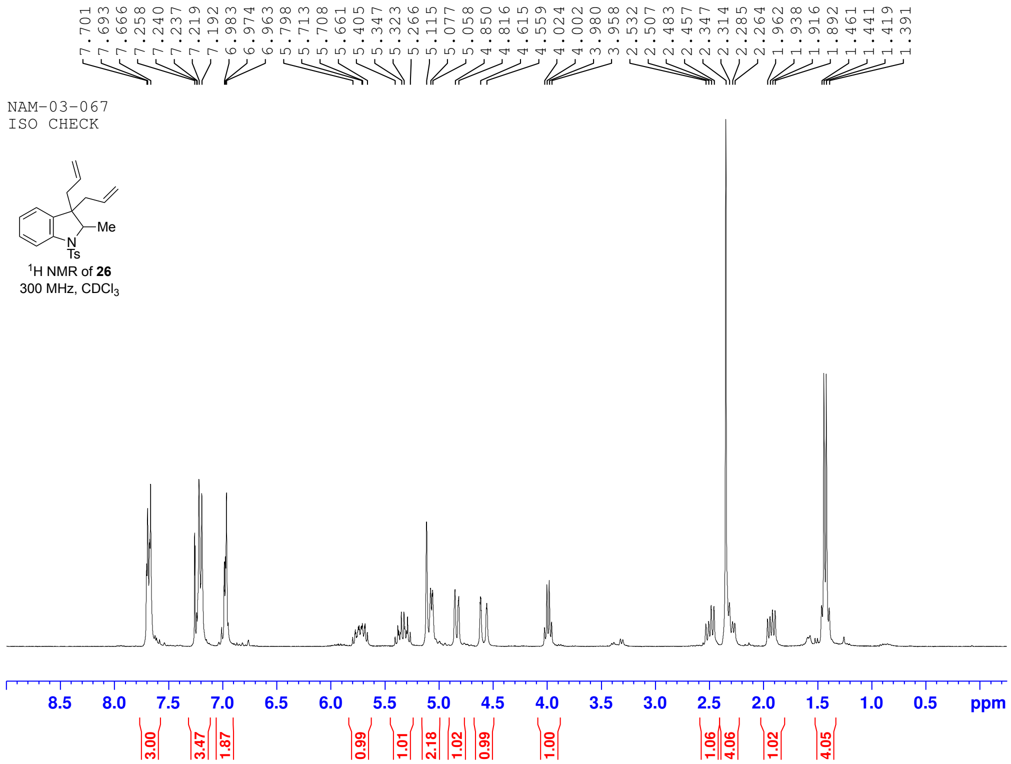
NAM-03-067
ISO CHECK
Me
N
Ts
1H NMR of 26
300 MHz, CDCl3
ppm

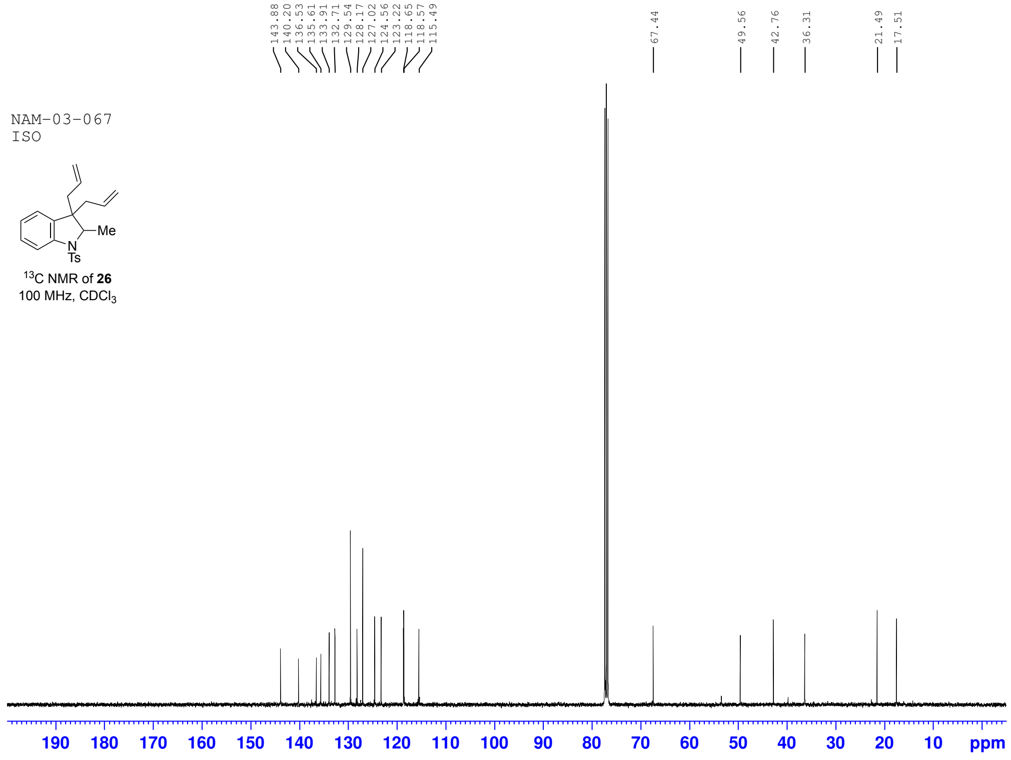
NAM-03-067
ISO
Me
N
Ts
13C NMR of 26
100 MHz, CDCl3
ppm

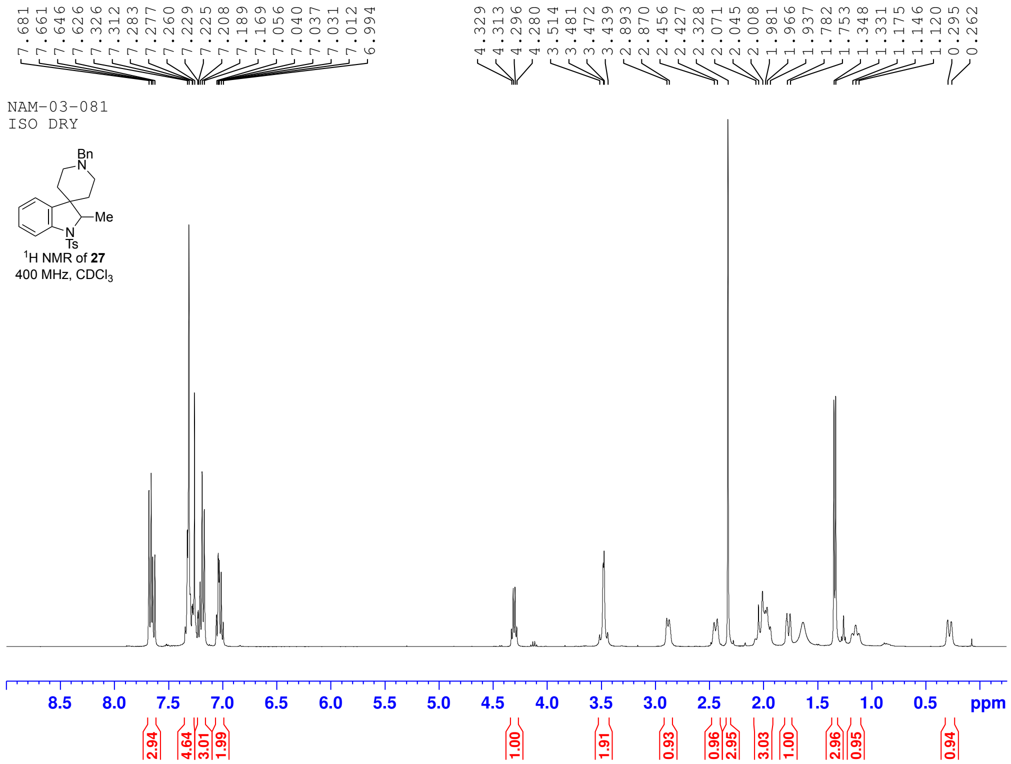
7.681
7.661
7.646
7.626
7.326
7.312
7.283
7.277
7.260
7.229
7.225
7.208
7.189
7.169
7.056
7.040
7.037
7.031
7.012
6.994
4.329
4.313
4.296
4.280
3.514
3.481
3.472
3.439
2.893
2.870
2.456
2.427
2.328
2.071
2.045
2.008
1.981
1.966
1.937
1.782
1.753
1.348
1.331
1.175
1.146
1.120
0.295
0.262
NAM-03-081
ISO DRY
Bn
N
Me
N
Ts
$^{1}$H NMR of 27
400 MHz, $CDCl_3$
8.5 8.0 7.5 7.0 6.5 6.0 5.5 5.0 4.5 4.0 3.5 3.0 2.5 2.0 1.5 1.0 0.5 ppm
2.94
4.64
3.01
1.99
1.00
1.91
0.93
0.96
2.95
3.03
1.00
2.96
0.95
0.94

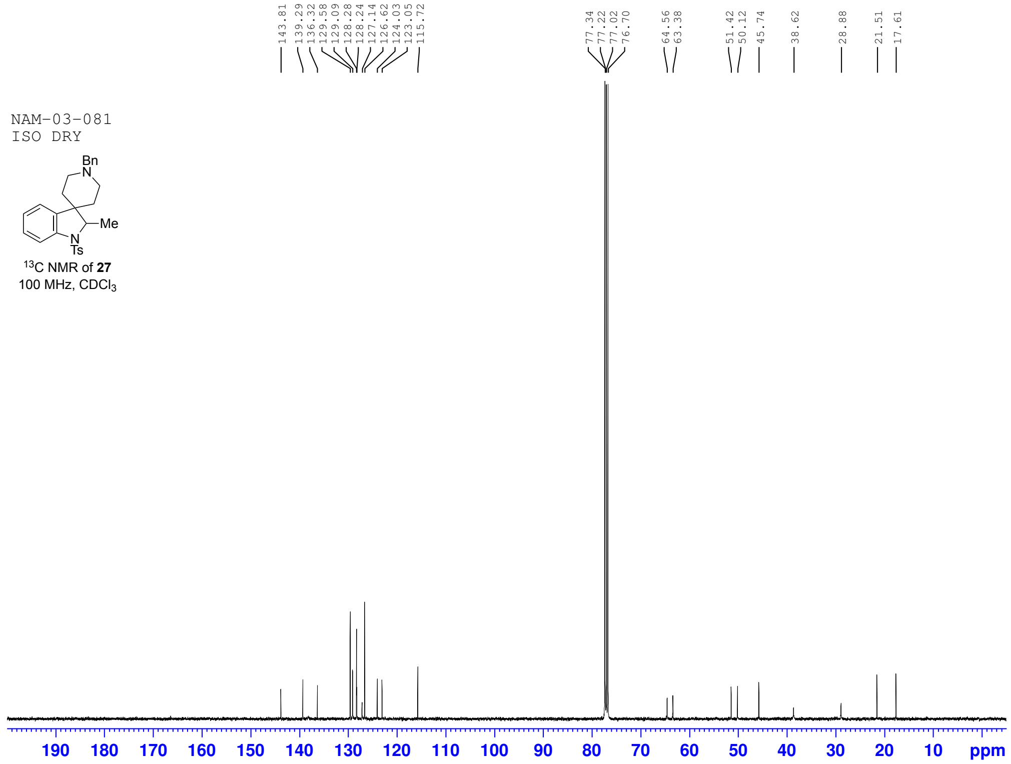
143.81
139.29
136.32
129.58
129.09
128.28
128.24
127.14
126.62
124.03
123.05
115.72
77.34
77.22
77.02
76.70
64.56
63.38
51.42
50.12
45.74
38.62
28.88
21.51
17.61
NAM-03-081
ISO DRY
Bn
N
Me
N
Ts
$^{13}$C NMR of 27
100 MHz, $CDCl_3$
190 180 170 160 150 140 130 120 110 100 90 80 70 60 50 40 30 20 10 ppm
